# Recent trends in therapeutic strategies for repairing endometrial tissue in intrauterine adhesion

**DOI:** 10.1186/s40824-021-00242-6

**Published:** 2021-11-24

**Authors:** Junyan Ma, Hong Zhan, Wen Li, Liqi Zhang, Feng Yun, Ruijin Wu, Jun Lin, Yangyang Li

**Affiliations:** 1 Zhejiang Provincial Key Laboratory for Precision Diagnosis & Treatment of Major Gynecological Diseases, Hangzhou, 310006 Zhejiang Province China; 2 Department of Gynecology and Obstetrics, Women’ s Hospital, Zhejiang University School of Medicine, Hangzhou, 310006 Zhejiang Province China

**Keywords:** Intrauterine adhesion, Biomaterial, Stem cell therapy, 3D printing, Electrospinning

## Abstract

Intrauterine adhesion (IUA) is a common gynaecological disease that develops from infection or trauma. IUA disease may seriously affect the physical and mental health of women of childbearing age, which may lead to symptoms such as hypomenorrhea or infertility. Presently, hysteroscopic transcervical resection of adhesion (TCRA) is the principal therapy for IUAs, although its function in preventing the recurrence of adhesion and preserving fertility is limited. Pharmaceuticals such as hormones and vasoactive agents and the placement of nondegradable stents are the most common postoperative adjuvant therapy methods. However, the repair of injured endometrium is relatively restricted due to the different anatomical structures of the endometrium. Recently, the treatment outcome of IUAs has improved with the advancement of hysteroscopic techniques. In particular, the application of bioactive scaffolds combined with tissue engineering technology has proven to have high therapeutic potential or endometrial repair in IUA treatment. Herein, this review has summarized past therapeutic strategies, including postoperative adjuvant therapy, cell or therapeutic molecular delivery therapy methods and bioactive scaffold-based tissue engineering methods. Therefore, this review presented the recent therapeutic strategies for repairing endometrium treatment and pointed out the issues of clinical concern to provide alternative methods for the management of IUAs.

## Introduction

**Fig. 1:**
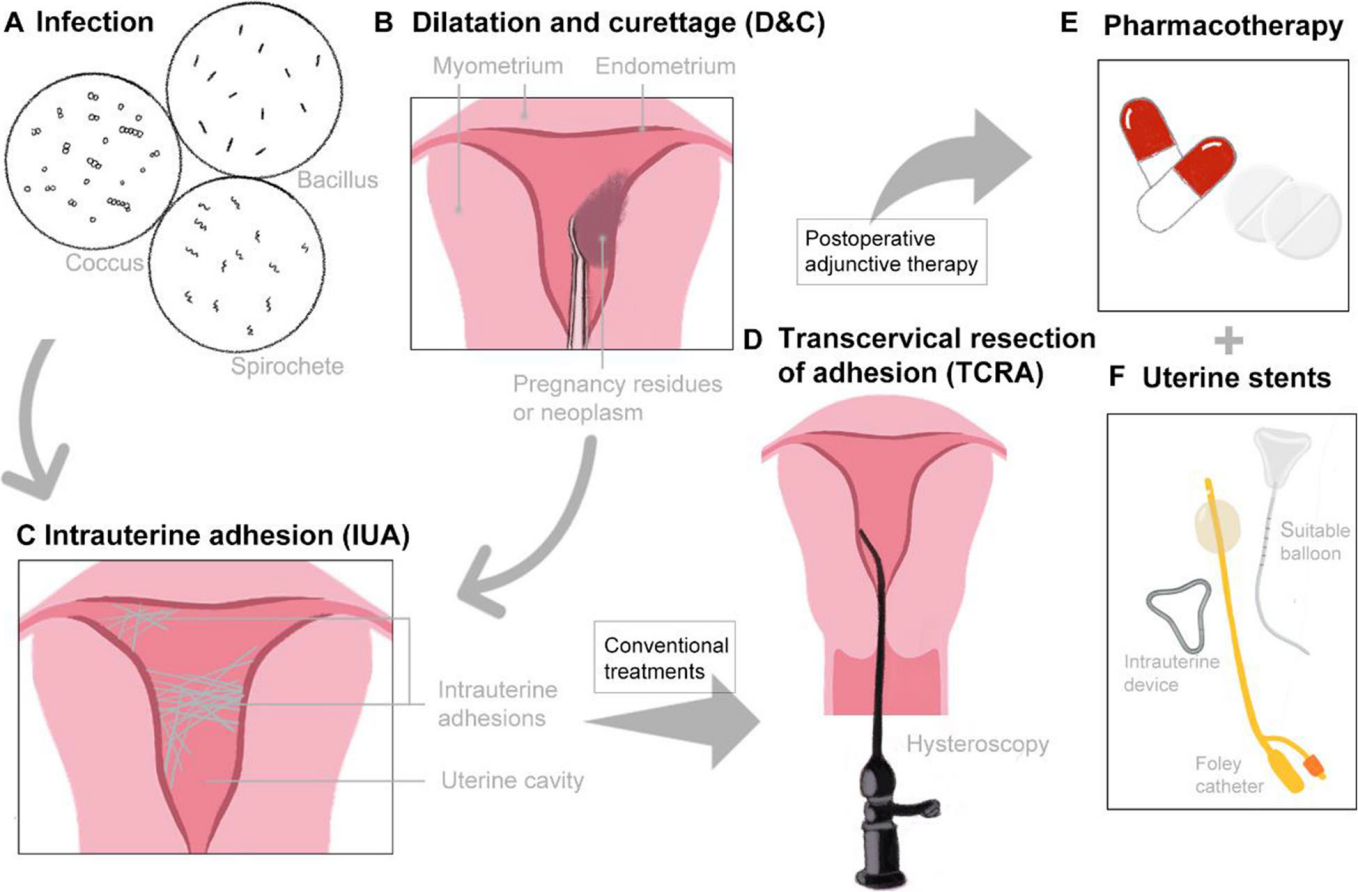
Aetiologies and Conventional treatments of IUA. (**A**) Endometrial infection and (**B**) intrauterine operations, such as D&C, are the main causes of (**C**) intrauterine adhesions (IUAs). The conventional treatments for IUAs are (**D**) hysteroscopic transcervical resection of adhesion (TCRA) followed by (**E**) drugs stimulating endometrial growth or/and (**F**) uterine stents to reserve the space of the uterus

Intrauterine adhesion (IUA), known as Asherman’s syndrome (AS), refers to endometrial injury induced by infection (Fig. 1A), trauma (Fig. 1B) and other reasons. IUA disease (Fig. 1C) may cause the uterine cavity and/or cervix to be completely or partially closed by fibrous adhesions, resulting in decreased menstrual volume, amenorrhea, periodic lower abdominal pain, secondary infertility, recurrent abortion, etc. [1, 2]. Some IUA patients with a successful pregnancy may even have symptoms such as placental adhesion, placental implantation and premature delivery [3]. All these clinical symptoms seriously affect the menstrual conditions and fertility requirements of women of childbearing age.

How does IUA happen? The basal layer of the endometrium is damaged, and then endometrial regeneration is obstructed and subsequently replaced by fibrous tissues without blood supply connecting the uterine walls [4]. The reason for the abnormal menstruation caused by IUAs is likely to be extensive damage to the endometrial basal layer and inability to regenerate the endometrium, which leads to reduced menstrual volume and even amenorrhea. Periodic lower abdominal pain may arise from adhesion in the cervix, which prevents menstrual blood from flowing smoothly out. Subsequently, the reflux of menstrual blood may contribute to blood accumulation in the fallopian tubes or even pelvic cavity, leading to lower abdominal pain. Secondary infertility may be relevant to the inability of sperm to enter the uterine cavity due to cervical canal or intrauterine adhesions and insufficient thickness of the endometrium to support the implantation of the fertilized egg. Patients with IUAs are prone to repeated abortion, which may be because the continued growth of the embryo is restricted by the narrow uterine cavity, and endometrial tissue and blood supply are insufficient for supporting the growth and development of the placenta. However, in patients with IUAs, the causes of further pregnancy-related complications, such as placental adhesion, placental implantation and premature delivery, have not been studied in depth. Although IUA is not life-threatening, it brings severe physiological and psychological troubles to a considerable number of women of fertility age, which should be considered by clinicians.

The treatment of IUAs aims to restore the normal uterine cavity shape, reduce the recurrence rate of adhesion, help repair the endometrium and improve the fertility of patients with IUAs. Most IUA patients without pregnancy requirements have no indications for treatment. Patients with minimal menstrual volume or even amenorrhea and repeated abortion demand fertility requirements. Surgical treatment and intimal repair are required. The standard clinical treatment scheme for IUAs is adhesiolysis under direct hysteroscopic visualization [5], and transcervical resection of adhesion (TCRA) (Fig. 1D) is the preferred surgical method for treating IUAs due to its perceptual intuition, accuracy and minimal invasion. It should be operated on under ultrasonic or laparoscopic monitoring to avoid perforation if necessary [6]. However, the surgical method can only separate the adhesions, and TCRA may even cause further damage to the residual endometrial tissue. The IUA recurrence rate of severe IUA patients after TCRA was as high as 20–62.5% [1]. Therefore, how to prevent postoperative adhesion in IUA patients and how to promote damaged endometrial regeneration to improve reproductive outcomes are the most urgent problems to be solved. Current routine treatments include postoperative hormone therapy (Fig. 1E) and placement of an IUD, a balloon, or anti-adhesion agents (Fig. 1F). Nevertheless, conventional methods in the clinic do not solve the problem of endometrial regeneration in severe cases and only result in limited effects and long treatment cycles. Moreover, there are potential risks of abnormal uterine bleeding, thrombosis, infection and other risks. The latest breakthrough in IUA therapy is the identification of appropriate biological tissues and functional bioactive scaffolds for intrauterine placement, aiming to promote or induce endometrial repair or provide an ideal environment for self-repair of endometrial cells to improve the reproductive function of patients. However, there still exist several challenges in the face of IUA regeneration and ideal therapeutic biomaterials to achieve effective treatments for IUA disease.

The present review offers a summary of available therapeutic strategies for repairing the endometrium in IUAs and highlights current gaps in research. We discuss the traditional and novel intervention methods for the prevention of IUAs, as well as emerging and developing therapeutics and delivery strategies that will most likely change the treatment regimen for better clinical effects. Finally, the challenges and future research expectations in repairing IUAs are also discussed. We believe this review would be beneficial for those who are working in areas related to the field of endometrial repair in IUAs.

## Postoperative adjuvant therapy methods

### Pharmacotherapy

The pharmacologic approach, which is the priority, is usually utilized in combination with other strategies in numerous studies [7]. Conventional therapies for IUA or thin endometrium include hormone therapy (large doses of oestrogen, progesterone), gonadotropin releasing hormone agonists, human chorionic gonadotropin, intrauterine perfusion of granulocyte colony stimulating factor (G-CSF), and therapeutic agents that improve blood flow perfusion, such as low-dose aspirin, vitamin E, and sildenafil citrate [8] **(**Table 1**)**. More details are introduced in the following sections.

**Table 1:** Pharmacotherapy in IUA

Medicine		Therapeutic effects	Side effects	Reference
Hormone	Estrogen	Improves endometrium repair (first-line)	Thrombosis; nausea	[9–11]
	Progesterone	Sequential use with estrogen; induces endometrial stromal cell proliferation and differentiation, and promotes endometrial decidualization	Chest pain, fever, dizziness, nausea	[12]
	Growth hormone	Plays a synergistic role with estrogen; improves the sensitivity of receptors on the endometrium to estrogen	Increased insulin resistance, edema, joint and muscle pain	[13]
Vasoactive agents	Aspirin	Inhibits endometrial fibrosis by suppressing the TGF-β1-Smad2/Smad3 pathway; promotes angiogenesis and enhances endometrial receptivity inhibits platelet aggregation, preventing microthrombus	Irritation of the stomach or gut, nausea, indigestion	[14–18]
	Nitroglycerine	Increases subendometrial blood flow velocity	Headache, dizziness, lightheadedness, nausea, and flushing	[19]
	Sildenafil	Enhances the uterine blood flow, improves ovulation success rate	Headaches, flushing and hypotension	[20, 21]
Traditional Chinese medicine	Herbs	Promotes blood circulation, removes blood-stasis	Unknow	[22]
	Acupuncture	Promotes blood circulation	Unknow	[23]
Antibiotics	Individualized	Active or prophylactic treatment of reproductive system infection	Antibiotic resistance,	[24, 25]

### Hormone therapy

Oestrogen or oestrogen plus progesterone (artificial cycle).

IUA patients usually suffer from extensive endometrial basal layer damage, and their endometrial tissue may be further damaged during hysteroscopy. Since it is well known that oestrogen can help regenerate the endometrium and accelerate wound repair, oestrogen therapy after TCRA seems quite indispensable. Related research has proven that postoperative oestrogen therapy for IUA patients can, to a certain extent, help repair the endometrium, improve menstrual volume, and reduce the occurrence of re-adhesion [9]. At present, hormone therapy is started immediately after TCRA, although there is no unified international standard on the dosage, dosage form and administration method of oestrogen.

The commonly used hormone therapy regimen is oral oestrogen for 21 days continuously, and progesterone can be added or not added during the latter 7–10 days according to the condition of endometrial repair. 2017 American Association of Gynecological Laparoscopists (AAGL) Practice Reports suggested using oral conjugated oestrogen 2.5 mg/d (with or without progesterone) for 2–3 cycles after TCRA [10]. A recent study also found that compared with large dose oral oestrogen (oestradiol valerate tablets 9 mg), medium dose oestrogen (oestradiol valerate tablets 4 mg) is more beneficial to reduce endometrial fibrosis after TCRA and improve endometrial acceptability [11], thus achieving the goal of reducing adhesion recurrence rate and improving reproductive functionality. A prospective randomized controlled study involving 121 patients revealed that there was no significant difference between oral oestradiol valerate tablets at 6 mg/d and 2 mg/d in the recurrence of adhesion after IUA surgery [26]. However, a higher dose of oestrogen will increase the likelihood of complications, such as thrombosis. As a result, it is not recommended to use a large dosage of oestrogen after TCRA. Oral oestrogen is preferred by the majority of clinicians at present, and there are relatively few studies on other applications, such as intrauterine or percutaneous administration and doses of oestrogen. Therefore, studies with large samples and long-term follow-up are needed to select the best route and dose of oestrogen and establish a unified standard.

#### Growth hormone

Growth hormone (GH), called somatotropin, has been extensively applied in regulating growth, metabolism, and reproduction [27, 28]. Considering the potential of improving oocyte quality and pregnancy rates, GH has been used in assisted reproductive technologies (ART) for several years [29, 30]. Recent studies provide evidence of extensive GH receptors in the endometrium, and they divert attention to the effect of GH on the endometrium [13]. GH and oestrogen play a synergistic role in improving the sensitivity of hormone receptors on endometrial cells and promoting protein synthesis and the metabolic function of the endometrium. Currently, GH is widely used to treat amenorrhea caused by unresponsive endometrium, which can significantly increase endometrial thickness and endometrial perfusion. Thus, the menstrual status can be improved and further enhance the endometrial receptivity and long-term pregnancy rate of the patients [31–34]. However, there is no clear research on the effect of long-term GH therapy.

### Increasing vascular perfusion to endometrium

Many research studies have indicated that various medications, which may increase vascular flow to the endometrium (such as aspirin [14], nitroglycerin [19], and sildenafil citrate [20]), have been utilized as ancillary treatments after lysis of IUAs. Further research is required to evaluate the possible adverse effects and exact efficacy given this off-label use. Moreover, traditional Chinese herbs [22] and acupuncture [23] are able to promote blood circulation and remove blood stasis, which may affect both ovarian and endometrial functions, resulting in a decisive alteration in oocyte maturation and endometrial epithelium receptivity. However, its safety and effectiveness also need to be further investigated.

### Aspirin

Aspirin is a type of acetylsalicylic acid drug. Low-dose aspirin (75 ~ 150 mg/d) can effectively suppress the synthesis of thrombin A2, thus inhibiting platelet aggregation, preventing microthrombus formation and improving blood circulation [14]. In addition, aspirin may inhibit endometrial fibrosis by suppressing the TGFβ1- Smad2/Smad3 pathway [15], and prevent vasoconstriction and platelet aggregation and improve local blood supply through its anti-inflammatory properties [16]. A recent study demonstrated that aspirin might promote endometrial proliferation and regeneration after TCRA in patients with severe IUA [17], while the side effects on reproduction are uncertain. At present, there are few reported studies on the application of aspirin in IUA treatment, and the specific mechanism remains to be further investigated. Meanwhile, whether aspirin has an impact on the female endocrine system is still unclear.

### Antibiotic therapy

Many previous reports have proposed that infectious factors play important roles in the pathogenesis of IUA. Bacterial (especially *Mycobacterium tuberculosis*), viral and other microbial infections are risk factors for adhesion formation inside the uterus. For example, there was a significant correlation between Chlamydia and/or Mycoplasma infection and IUA [24]. The incidence of reproductive tract tuberculosis is high in IUA patients [25]. Therefore, prophylactic or active treatment of reproductive system infection before uterine cavity surgery is important to prevent postoperative IUA formation. It might be necessary to treat patients undergoing surgical lysis of IUAs with preoperative or intraoperative antibiotics, and postoperative antibiotic therapy may also continue in certain cases.

### Alternative uterine stent/non-degradable scaffold

**Fig. 2:**
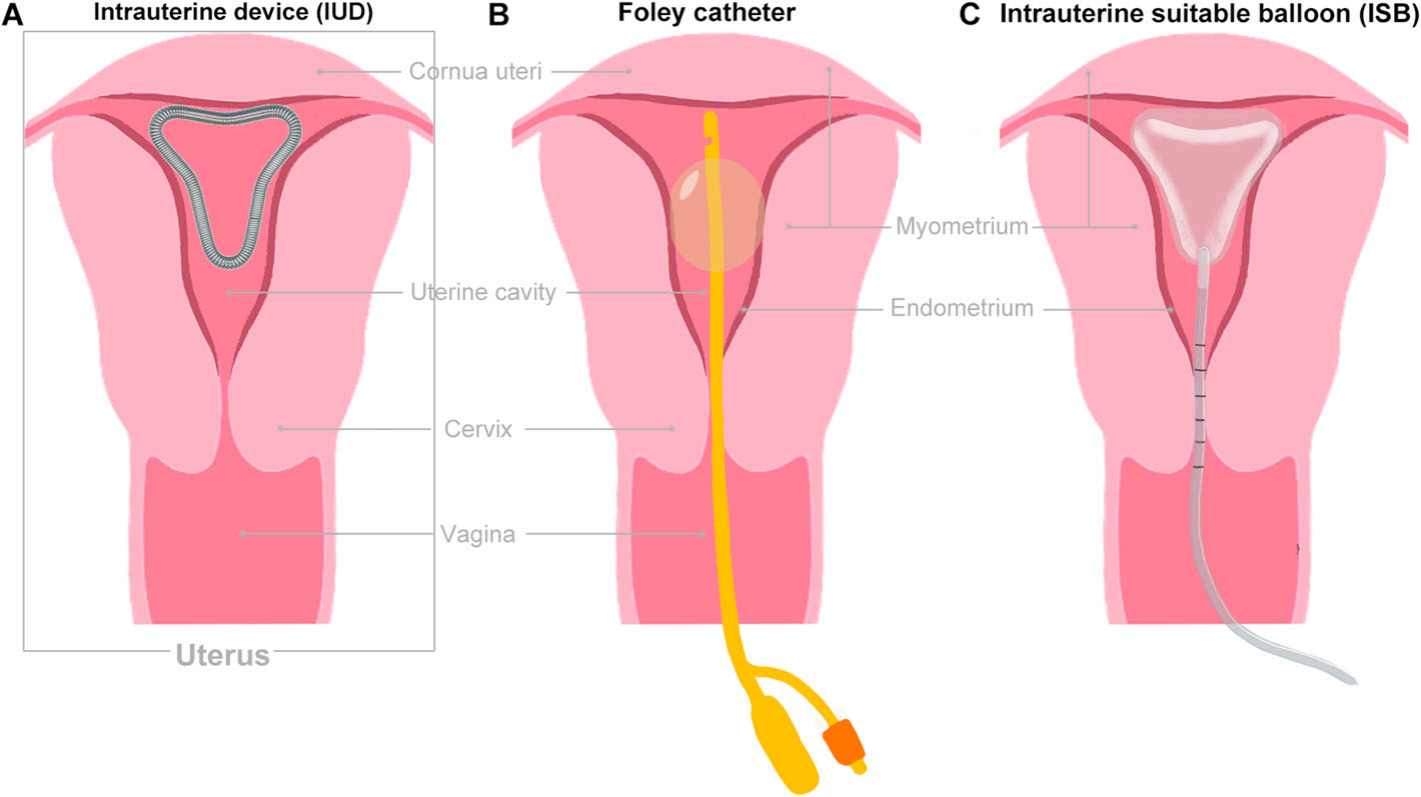
Alternative uterine stent/non-degradable scaffold. (**A**) An intrauterine device (IUD), (**B**) a Foley catheter, or (**C**) an intrauterine suitable balloon (ISB) will be placed in the uterus as the stent to support the uterine cavity

Although the role of alternative stents in preventing the recurrence of IUAs and subsequent fertility outcomes is unclear, they are frequently used after hysteroscopic adhesiolysis. There are three types of isolation barriers: intrauterine devices (IUDs) **(**Fig. 2A**)**, Foley catheters **(**Fig. 2B**)**, and intrauterine suitable balloons (IBSs) **(**Fig. 2C**)**, which are commonly placed in the uterine cavity after separation of IUAs [35]. The specific choice is primarily dependent on the preferences of the surgeons and the different degrees and types of adhesions.

#### Intrauterine device (IUD)

The intrauterine device (IUD) was originally designed only for contraception, although now it is commonly used in clinical practice. According to the different types and grades of adhesion, the IUD (T shape, O shape or uterine shape, etc.) is selected individually to support the uterine cavity to a proper space and separate the surgical wounds. Placement of an IUD after TCRA is carried out to prevent recurrence of adhesions and restore menstrual volume [36]. A systematic review revealed that IUD placement after TCRA somewhat contributes to the recovery of menstrual volume in IUA patients [37]. Meanwhile, it is necessary to combine it with other corresponding treatments, such as hormone therapy, sodium hyaluronate injection or amniotic membrane placement, to maximize the benefits of patients. However, the therapeutic effects of traditional IUDs are not always ideal, attributed to the limited surface of the device and incomplete separation of the anterior and posterior walls, especially in the centre of the uterine cavity. Thus, the possibility of re-adhesion outside the IUD may also occur. Some researchers thought T-shaped IUD was not suit to prevent adhesion reformation because of the limited surface, and IUD containing copper may lead to an excessive inflammatory reaction The uterine-shaped IUD consists of a stainless steel coiled wire with copper added inside, and it was preferable compared to the former two on account of releasing anti-inflammatory agent [38]. Moreover, if the IUD is left inside of the uterine cavity for too long, aseptic inflammation will be induced due to foreign body stimulation, which may result in uterine cavity infection and bleeding and thus lead to fibrosis, IUD incarceration and even uterine perforation. There is also uncertainty about the type and duration course of IUD to be used in IUA.

#### Foley catheter

Foley catheters were initially used for urethral catheterization, but they have recently been widely used in other clinical applications. The Foley catheter acts as a physical barrier separating the surgical wound and supporting the entire uterine wall to prevent recurrence of adhesion. Compared with IUDs, Foley catheter placement after TCRA appears to be more effective in reducing complications, such as infection, and can help patients recover from reduced menstruation and adhesion due to the larger wound space [39]. Someone noted the fact that it was more effective to place a Foley catheter than an IUD, while the intrauterine balloon might be more helpful in preventing IUA [7].

#### Intrauterine suitable balloon (ISB)

A “heart”-shaped intrauterine suitable balloon (ISB, Patent number: 201420679083.7) was designed to reduce bleeding after intrauterine surgery. Currently, ISB is commonly used to reduce wound secondary adhesion and the IUA recurrence rate after TCRA. An ISB is inserted inside of the uterus, and then an appropriate volume of water is injected into the balloon to separate and sustain the anterior and posterior walls of the uterus and bilateral uterine corners. A retrospective study of 150 patients with moderate-to-severe IUAs demonstrated that intrauterine placement of an ISB followed by TCRA was more effective than a Foley balloon in reducing postoperative AFS scores, especially in preventing the recurrence of severe IUAs (25.0% vs. 35.1%) [40]. However, insufficient water injection in the balloon could not be effectively supported, and redundant water injection would lead to excessive pressure in the uterine cavity and excessive oppression of the endometrial tissue, resulting in ischaemia and necrosis and thus affecting its regeneration and repair. To date, there is no universal agreement on the amount of water injected into the balloon and the retention time of the balloon. Meanwhile, more clinical studies on this are essential to substantiate the findings.

### Biological barriers

#### Amniotic membrane transplantation (AMT)

**Fig. 3:**
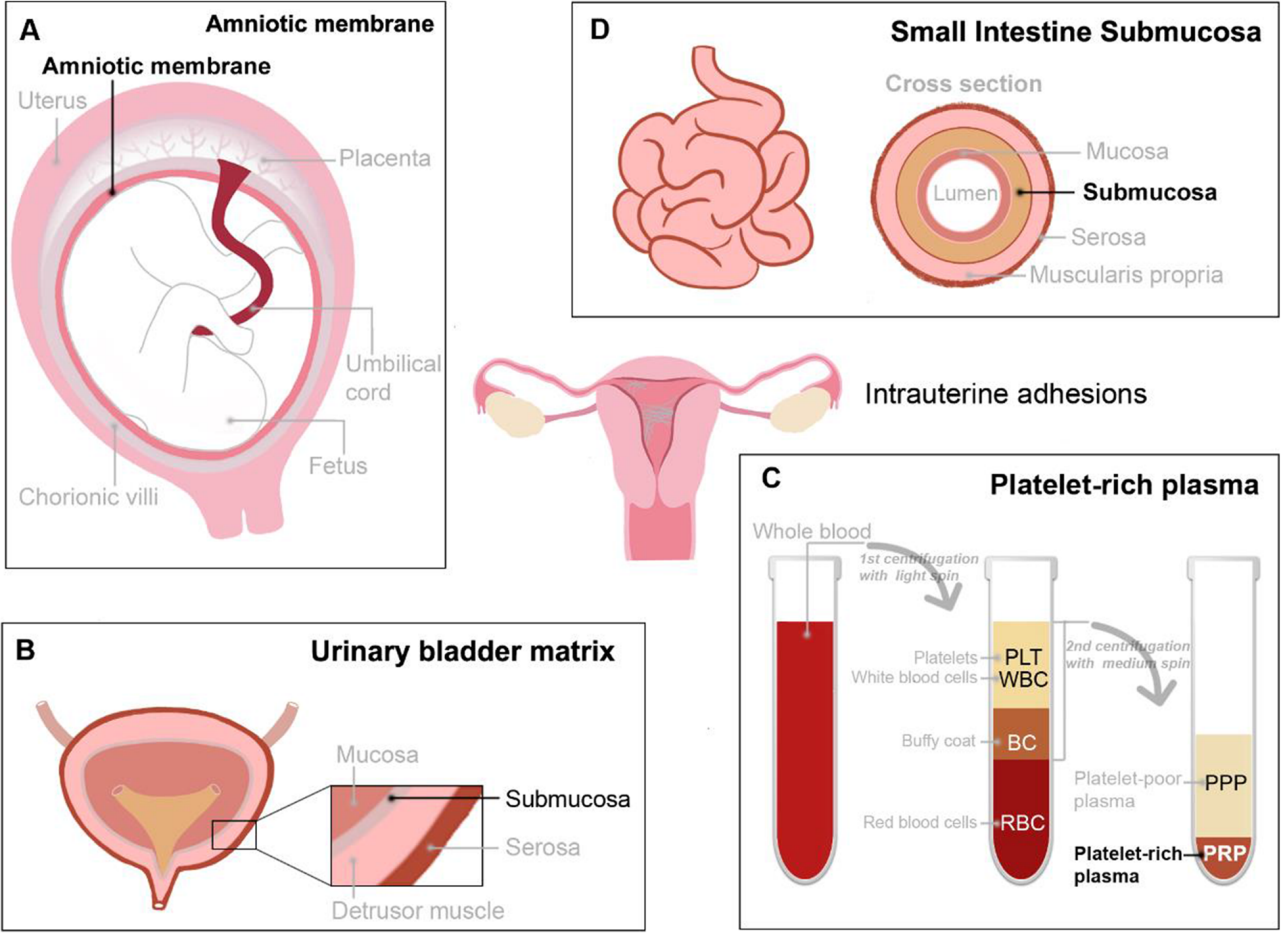
Biological barriers. (**A**) Human amniotic membrane (AM) originates from placenta. (**B**) Urinary bladder matrix (UBM) is a derived extracellular matrix (ECM) from porcine bladder with an intact basement membrane. (**C**) Platelet-rich plasma (PRP) was extracted from fresh whole blood after two centrifugations. (**D**) Small intestine submucosa (SIS) is applied as a decellularized matrix derived from small intestinal segments of porcine

**Fig. 4:**
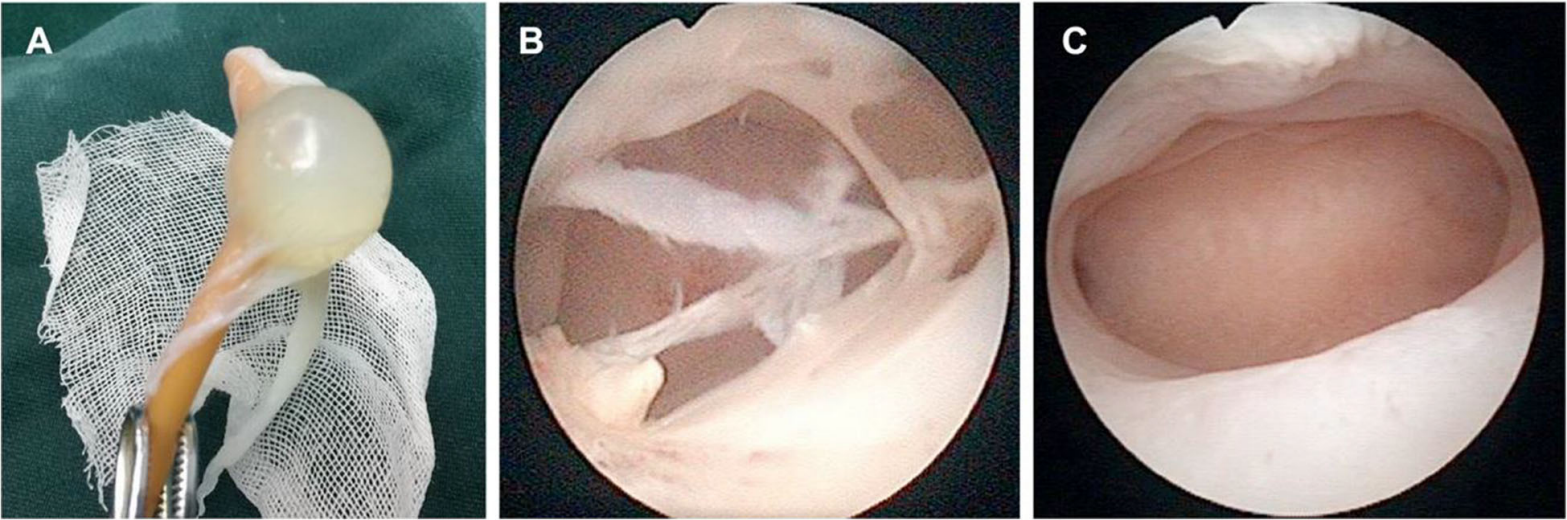
Amniotic membrane transplantation prevents reformation of intrauterine adhesions [44]. (**A**) Amnion membrane over Foley balloon. (**B**) Hysteroscopy (4 weeks after operation) showed some amnion membrane tissue remaining in the uterine cavity. (**C**) Hysteroscopy (12 weeks after operation) showed a uterine cavity with normal endometrium and no amnion membrane tissue left. Reprinted with permission from Ref. [44]

The amniotic membrane (AM) **(**Fig. 3A**)** originates from the placenta and is formed by the differentiation of trophoblast cells without nerves, blood vessels or lymph vessels. The epithelial cells of AM not only secrete glycoproteins and collagen fibres, but also a variety of growth factors and cytokines such as epidermal growth factor (EGF), vascular endothelial growth factor (VEGF), keratinocyte growth factor, basic fibroblast growth factor (bFGF), transforming growth factors alpha and beta (TGFαand TGFβ), interleukin-8 (IL-8), angiogenin, dipeptidyl peptidase IV (DPPIV/CD26), type 1 plasminogen activator inhibitor (PAI-1), insulin-like growth factors (IGF) and their binding proteins. These variety of bioactive factors promote the growth and migration of epithelial cells. Hyaluronic acid is abundant in the extracellular matrix of the AM, which facilitates cell movement and exhibits anti-inflammatory and immunosuppressive properties [41]. The human AM contains abundant human amniotic mesenchymal stromal cells (hAMSCs), which are a type of pluripotent stem cell that has the potential to induce differentiation into endometrial cells [42]. Amer et al. first used the AM over an inflated balloon of a Foley’s catheter for 2 weeks after hysteroscopic adhesiolysis in moderate and severe IUA patients, and AMT seemed promising in decreasing adhesion recurrence to promote endometrial repair [43]. A retrospective cohort study also proved the safety and efficacy of amnion grafts in preventing adhesion recurrence during the second-look and third-look hysteroscopies after 1 and 3 months. However, there was no significant effect on increasing pregnancy rate (Figs. 4, [44]). While, more recently, a comprehensive meta-analysis (four studies and 300 patients) of the application of AMT in the treatment of IUAs indicated that AMT is able to effectively improve menstrual volume in IUA patients after TCRA, but AMT did not affect the rates of IUA recurrence, pregnancy or spontaneous abortion [45]. Substantial clinical randomized controlled studies and long-term follow-up are still needed to confirm the efficacy of AMT in the treatment of IUA.

#### Urinary bladder matrix (UBM)

**Fig. 5:**
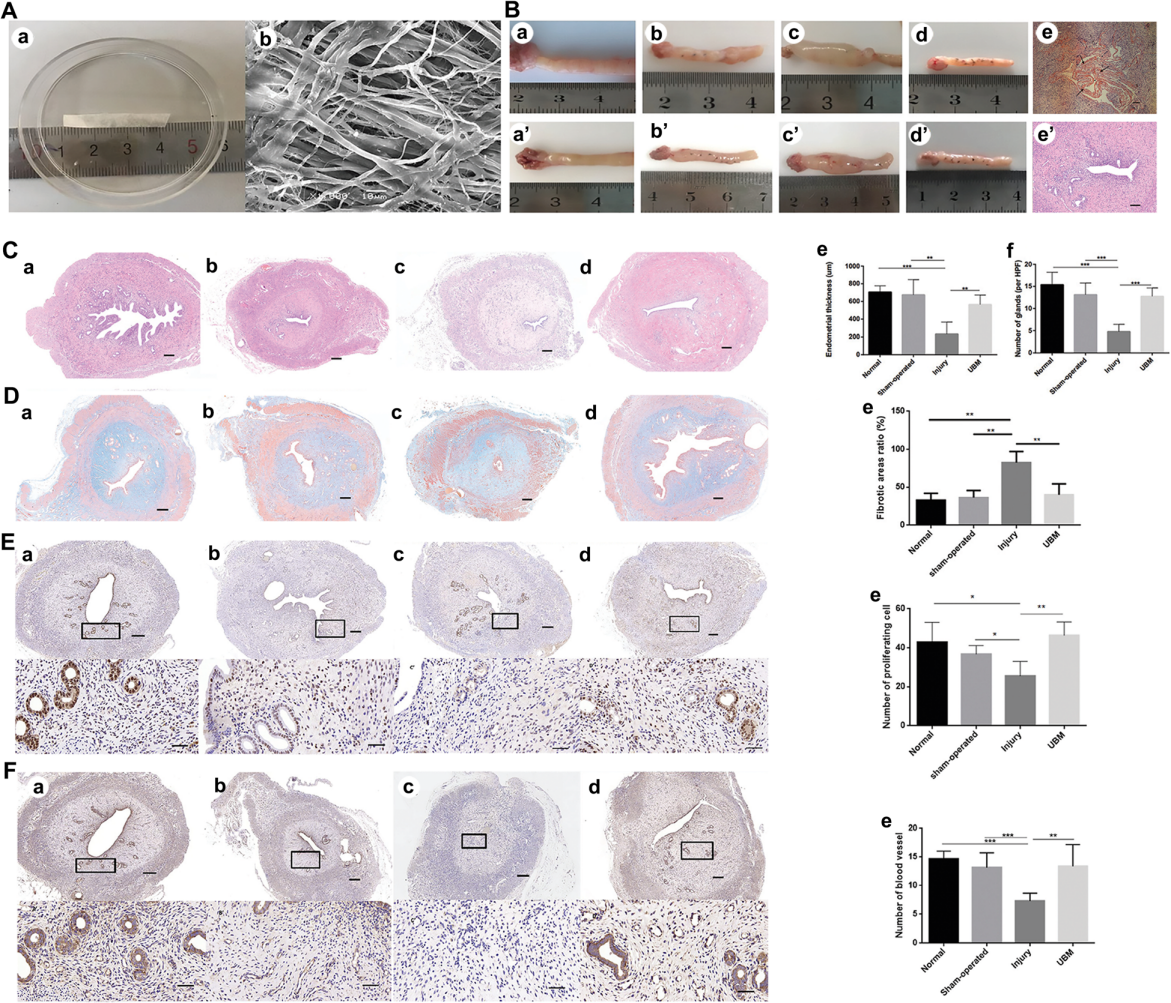
Urinary bladder matrix (UBM) scaffolds improve endometrial regeneration in a rat model [51]. (**A**) (a) Appearance and structure of the UBM, 3.0 cm in length, 0.6 cm in width, and 0.1 cm in thickness. (b) A scaffold with a 1000 μm pore size shown by scanning electron microscopy. (**B**) Gross view and histological structure of reconstructed uterine horns at 2 weeks (a − e) and 4 weeks (a’ − e’) postoperation in the normal group (a and a’), the sham-operated group (b and b’), the injury group (c and c’) and the UBM group (d and d’). Bar:100 μm. (**C**) Histological structure of the uterine horns. (**D**) UBM transplantation reduces injury-induced fibrosis. Masson’s trichrome staining of the collagen. (**E**) UBM transplantation increased cell proliferation. Immunohistochemical staining of Ki67. (**F**) UBM transplantation increased blood vessel distribution in the endometrium. Immunohistochemical staining of blood vessels (vWF) in the endometrium at 4 weeks after surgery (a to d) in the normal group (a), the sham-operated group (b), the injury group (c) and the UBM group (d). (a-d, Bar: 200 μm), (a’-d’: Bar: 50 μm). **p < 0.01 and ***p < 0.001. Reprinted with permission from Ref. [51]

Urinary bladder matrix (UBM) **(**Fig. 3B**)** is a derived extracellular matrix (ECM) from porcine bladder with an intact basement membrane, which can provide structural support and regulate cell adhesion, migration, and proliferation [46]. UBM possess plenty of advantages in tissue repair. Firstly. Urinary bladder matrix contains 95% ingredients for structural proteins, including IV collagen, which is lacking in other organizations [47]. Second, it contains 5% bioactive components, rich in adhesive proteins, glycans (hyaluronic acid/chondroitin sulfate, etc.), and active factors that support and regulate cell growth and differentiation [48]. Third, it is the “soil” and “signal” of tissue regeneration. Compared with ECM derived from other tissues, such as small intestinal submucosa, acellular dermal matrix, cholecyst-derived extracellular matrix, acellular pericardium, UBM is closer to natural immunity, has natural resistance to infection, ideal mechanical properties, higher biological activity and tissue regeneration induction [49]. After implantation of UBM in vivo, the proliferation and differentiation of endometrial cells are promoted, and original specific tissues are formed at the damaged sites to realize partial functional recovery [50]. A recent study transplanted UBM into the uterine horns of IUA rats resulted in endometrial regeneration, including thick endometria with increased glands, decreased fibrotic areas and improved endometrial receptivity. Compared to injury group, increased anti-inflammatory cytokines (bFGF) and endometrial receptivity factors (leukemia inhibitory factor and integrin αVβ3), and decreased proinflammatory cytokines (tumor necrosis factor α) were found in UBM group (Fig. 5) [51].

#### Platelet-rich plasma (PRP)

**Fig. 6:**
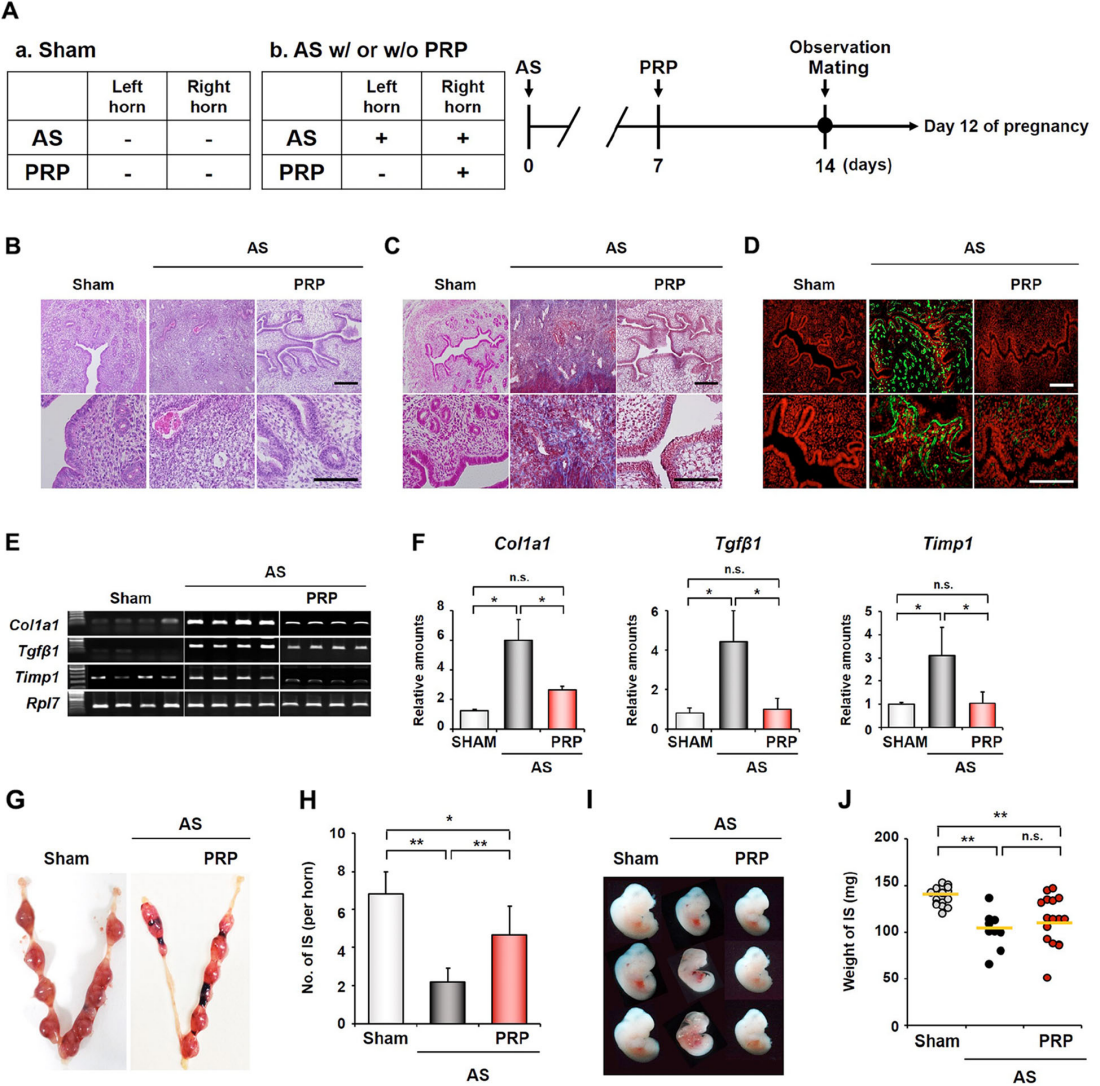
Human platelet-rich plasma (PRP) infusion repairs the damaged endometrium and improves implantation outcomes in Asherman’s syndrome (AS) mice [58]. (**A**) PRP was injected only into the right horn at 7 days after inducing injury to the bilateral uterine horns. At 14 days, some mice were sacrificed, and the uterine horns were prepared for analysis. The other mice were mated with fertile, healthy males. (**B**) HE staining of the endometrial tissues to evaluate morphologic structures. (**C**) Masson’s trichrome staining to evaluate collagen deposition (blue). (**D**) Immunofluorescence staining of collagen type 1A (Col1a1). (**E**) PCR results and (**F**) analyses of the expression of fibrosis-related factors (Col1a1, Tgfβ1, and Timp1). (**G**) Gross morphology of the implantation sites (IS) in AS mice with or without PRP. (**H**) The number of ISs in the PRP-treated horns of AS mice. (**I**) Photographs of embryos isolated from the IS on Day 12 of pregnancy. (**J**) A graph of the weights of IS between the AS group and the PRP-treated AS group on Day 12 of pregnancy. (B-D, up and down bar: 100 and 200 μm. Reprinted with permission from Ref. [58]

Platelet-rich plasma (PRP) **(**Fig. 3C**)** can be extracted from fresh whole blood, whose platelet content is 3–5 times that of ordinary plasma. PRP contains high concentrations of growth factors such as EGF, VEGF, platelet-derived growth factor (PDGF), TGF, certain proteins and peptides (such as fibrinogen, fibronectin, osteonectin, osteocalcin, and platelet reactive protein), and certain chemokines and cytokines [52, 53]. With increasing research and understanding, PRPs have been mainly used for bone, tendon, wound, scar, skin and other tissue regeneration and repair [53–56] and have now been gradually applied in the field of reproduction. High concentrations of growth factors and cytokines in autogenous RPR can stimulate mitosis and proliferation of endometrial cells or endometrial stem cells and subsequently activate the endocrine paracrine pathway to improve the endometrial response and promote embryo implantation and pregnancy in frozen embryo transfer cycles [57]. Meanwhile, PRP can reduce the fibrosis caused by endometrial damage, increase the local anti-inflammatory effect of the uterine cavity, and improve endometrial receptivity **i**n IUA murine models **(**Fig. 6**)** [58, 59]. In addition, in vitro experiments confirmed that activated PRP could promote the migration and proliferation of endometrial epithelial cells and stromal fibroblasts different human endometrial cells such as epithelial cells, stromal fibroblasts and Ishikawa endometrial adenocarcinoma cells, to facilitate tissue regeneration [60, 61]. PRP has also been used in the clinical management of poor endometrial growth or IUAs, and intrauterine PRP infusion for female patients with thin endometrium and poor response can lead to successful endometrial expansion and pregnancy when compare with the negative control [60, 61].

#### Small intestine submucosa (SIS)

**Fig. 7:**
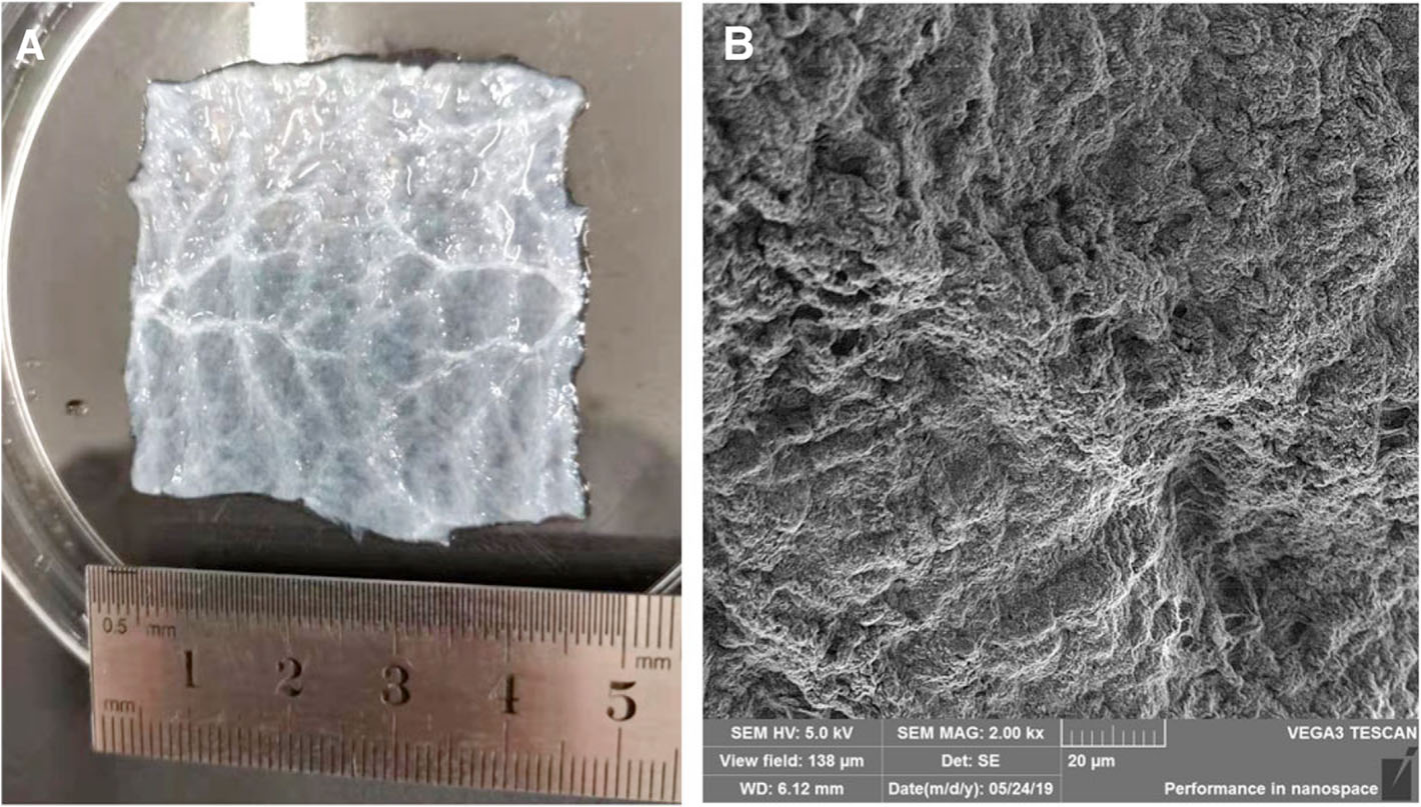
Modification of the small intestine submucosa (SIS) was used in tissue engineering. (**A**) The macroscopic surface morphology of the SIS after decellularization and (**B**) the SEM image of the SIS after decellularization. Reprinted with permission from Ref. [62]

Small intestine submucosa (SIS) **(**Fig. 3D**)** can be applied as a decellularized matrix derived from small intestinal segments of pigs. With preserved bioactive factors and growth factors, SIS has gained much attention in tissue engineering and subsequent clinical applications as a highly supportive scaffold **(**Fig. 7**)** [62]. More recently, in vivo research on the application of porcine SIS in the treatment of IUAs demonstrated that SIS can change the receptive factors (higher expressions of uteroglobin and HOXA10) and significantly increase endometrial thickness and reduce the percentage of fibrotic areas in IUA rat modes [63]. SIS was considered to promote endometrial regeneration and improve endometrial receptivity. Thus, SIS might be a potential biological barrier for the formation of IUAs.

### Polymer based biomaterials and their derivatives

Anti-adhesion agents are mechanical barriers that can effectively separate the walls of the uterine cavity **(**Table 2**)**. Sodium hyaluronate and chitosan are commonly used bioactive materials for antiadhesion applications at present. These medical high polymer polysaccharides possess good biocompatibility and biodegradable performance, can be evenly dispersed by the contraction of the uterine cavity itself and act as a barrier to block damaged wounds, making the endometrium less affected by inflammation and scar formation during the repair process. Intrauterine application of anti-adhesive gel is effective as primary and secondary preventions. For example, there are three gels that have been used: auto-cross-linked polysaccharide hyaluronic acid (ACP), hyaluronate carboxymethylcellulose membrane (CH) and polyethylene oxide-sodium carboxymethylcellulose (POC) [91].

**Table 2:** Polymer based materials and their derivatives

Polymer materials	Advantages	Disadvantages	Reference
Hyaluronic acid	Good biocompatibility, fluidity and adhesion	High liquidity, fast absorption rate, short retention time	[64–67]
Chitosan	Good biocompatibility, biodegradability, non-antigenicity, non-toxicity, antibacterial, hemostatic activity	Weak mechanical strength, hard and brittle	[68, 69]
Cellulose (Interceed)	good biological description, easy to be absorbed by the body, and has no obvious toxic effect.	Inconvenient placement	[70–73]
Collagen / gelatin	Good biocompatibility and low antigenicity Biocompatible Biodegradable Cell adhesion Integration with new tissue matrix	High degradation rate, poor mechanical properties No inherent rigidity Potential for antigenicity	[74–78]
Alginate	Biocompatible Biodegradable Stable in the form of hydrogel	Difficulty of purification Potential of cytotoxicity	[69]
Silk fibroin	Easy to get, cheap, Good biocompatibility, slow Biodegradation rate and low Inflammatory response Biocompatible Biodegradable	Poor mechanical properties and toughness Restricted length Fast degradation	[79]
Poly ε-Caprolactone (PCL)	Good cytocompatibility Histocompatibility, Biodegradability and elasticity	Excessive flexibility and elasticity	[80]
Polylactide (PLA)	Biodegradable Excellent mechanical properties and chemical stability	Rapid degradation rate Aseptic inflammation caused by the acidic degradation products;	[81]
Poly (lactic-co-glycolic acid (PLGA)	Good biocompatibility, degradation, processing, and plasticity Biodegradable and biocompatible generally low toxicity, possessing high capacity for drug loading, and having potential for a range of rates of degradation and drug release	Aseptic inflammation caused by the acidic degradation products; Poor cell adhesion	[82, 83]
Heparin-Proloxamer	Low toxicity, high affinity, slow release, fixed point release anticoagulant and thrombolytic properties temperature-sensitive		[84–89]
Poly (glycerol sebacate), PGS	Favors the attachment and growth of rat BMSCs Prolongs the retention time of BMSCs Directly differentiate into endometrial stromal cells after transplantation of PGS/BMSCs constructs		[90]

With the rapid development of nanotechnology, nanoparticle-based drug delivery systems (DDSs) have been used for tissue injury repair application [92]. These nanoparticle-based DDSs possess good penetration and retention ability and can provide therapy at the damaged site. Meanwhile, high drug loading efficiency and controlled manner may be the additional advantages. Nanoparticle based DDSs can be made from a variety of materials, including synthetic polymer, biopolymers, and naturally based materials such as proteins. Hence, these polymer-based DDSs possess good biodegradability and biocompatibility, which may be a safer, more reliable and less risky treatment compared to surgical treatment.

#### Hyaluronic acid (HA)

At present, hyaluronic acid (HA) gel has been widely utilized for lubrication in orthopaedic knee arthritis, prevention of adhesion and repair of peripheral nerve injury in various surgeries. Due to the characteristics of crude fluid and lubrication, HA can be used in separated endometrial wounds directly as a good physical barrier to prevent IUAs [64]. HA gel can be retained in the uterine cavity for approximately 72 h. It can act as a mechanical and physical barrier and thus reduces capillary bleeding in the uterus, improves the homeostatic environment in the uterine cavity, and effectively reduces the friction of the rough wound surface of viscera. A prospective RCT involving 132 patients demonstrated that the IUA recurrence rate (10.44%) of 10 ml auto crosslinked sodium hyaluronate (ACP) gel injected after a single surgically remediable intrauterine lesion (myomas, polyps or uterine septa) was lower than that of the untreated hysteroscopic group (26.15%) [65]. Another prospective study reported that a new crosslinked hyaluronan (NCH) gel was able to reduce IUA formation after intrauterine operation even in women who undergo curettage in the second trimester [66]. However, the therapeutic effect of HA gel used alone for IUA separation is not obvious. A systematic review study demonstrated that HA gel was less effective than IUDs and balloon stents. The reason may be that the coagulation time of HA gel in utero is too long, and the HA gel will quickly flow out of the uterine cavity after intrauterine separation; therefore, it cannot form a barrier for long [67].

#### Chitosan

Medical chitin is a synthetic high molecular polysaccharide substance that poses good biocompatibility, degradability and biological activity. At present, it has been frequently used to prevent intestinal adhesion after abdominal surgery, re-adhesion after reoperation of fallopian tubes and re-adhesion after hysteroscopic surgery. The mechanism of chitosan in preventing adhesion may be the following reasons [68]. First, medical chitin-gel materials can be skilfully made to promote fibrocyte growth and inhibit the formation of accumulated scar tissue. Subsequently, the growth of epidermal cells and endothelial cells is promoted. Thus, the repair of normal physiological tissue can be facilitated, and the probability of tissue adhesion can be hindered. Second, chitosan possesses the function of local haemostasis and inhibits bacterial growth. Hence, it can reduce the chance of infection after IUA separation. Third, due to the lubrication and physical barrier, chitosan takes approximately 3 weeks to decompose and be absorbed in the uterine cavity. The placement of medical chitosan in the uterine cavity after TCRA is conducive to the repair of the endometrium. Therefore, combination with oestrogen-assisted therapy will enhance the effect of repairing the endometrium and preventing re-adhesion.

#### Oxidized regenerated cellulose (Interceed)

INTERCEED™ (TC7) Absorbable Adhesion Barrier is a material of oxidized regenerated cellulose. Interceed is a soft woven fabric made of oxidized regenerated fibre. It is the first product approved by the FDA for postoperative adhesion prevention. Interceeds possess good biological descriptions, are easily absorbed by the human body, and have no obvious toxic effects. It is primarily indicated as an adjuvant in gynaecologic pelvic surgery for reducing the incidence of postoperative pelvic adhesions. An in vivo investigation of a rabbit model proved that combination therapy with Interceed and oestrogen was able to reduce IUA recurrence and tissue fibrosis and improve endometrial receptivity [70]. At present, it has been widely applied in the abdominal cavity, tendon, pelvis and other surgical sites in orthopaedics, obstetrics and other general surgeries [71, 72].

Interceed anti-adhesion membranes have been preliminarily explored to prevent postoperative intrauterine adhesion [73]. Before the operation, it can be trimmed into different sizes and shapes according to the surgeon’s needs to adapt to different sizes of the uterine cavity. Ultrasound can be utilized to confirm whether the Interceed is placed in the uterine cavity for at least 1 month. The fixed Interceed is able to form an effective physical barrier, prevent the re-contact of intrauterine surgical wounds, and provide space for endometrial hyperplasia and repair.

The mechanisms of its prevention of adhesion are summarized below [72, 93]. First, Interceed products will form a physical barrier or a protective film. Second, the function of macrophages can be affected, and thus, the state of the inflammatory response will be different. Third, it releases protease to degrade fibrin by activating fibrinolytic activity. Fourth, it will help to increase the endometrial glands, reverse endometrial fibrosis, improve endometrial receptivity, and promote endometrial repair to some extent. However, it is still difficult to place the incision into the uterine cavity. Therefore, further consideration should be given to improving the Interceed placement in clinical application.

## Cell or molecular delivery therapy strategies

### Role of stem cells in the repair of endometrial injury

#### Differentiation into endometrial cells and/or vascular cells

**Fig. 8:**
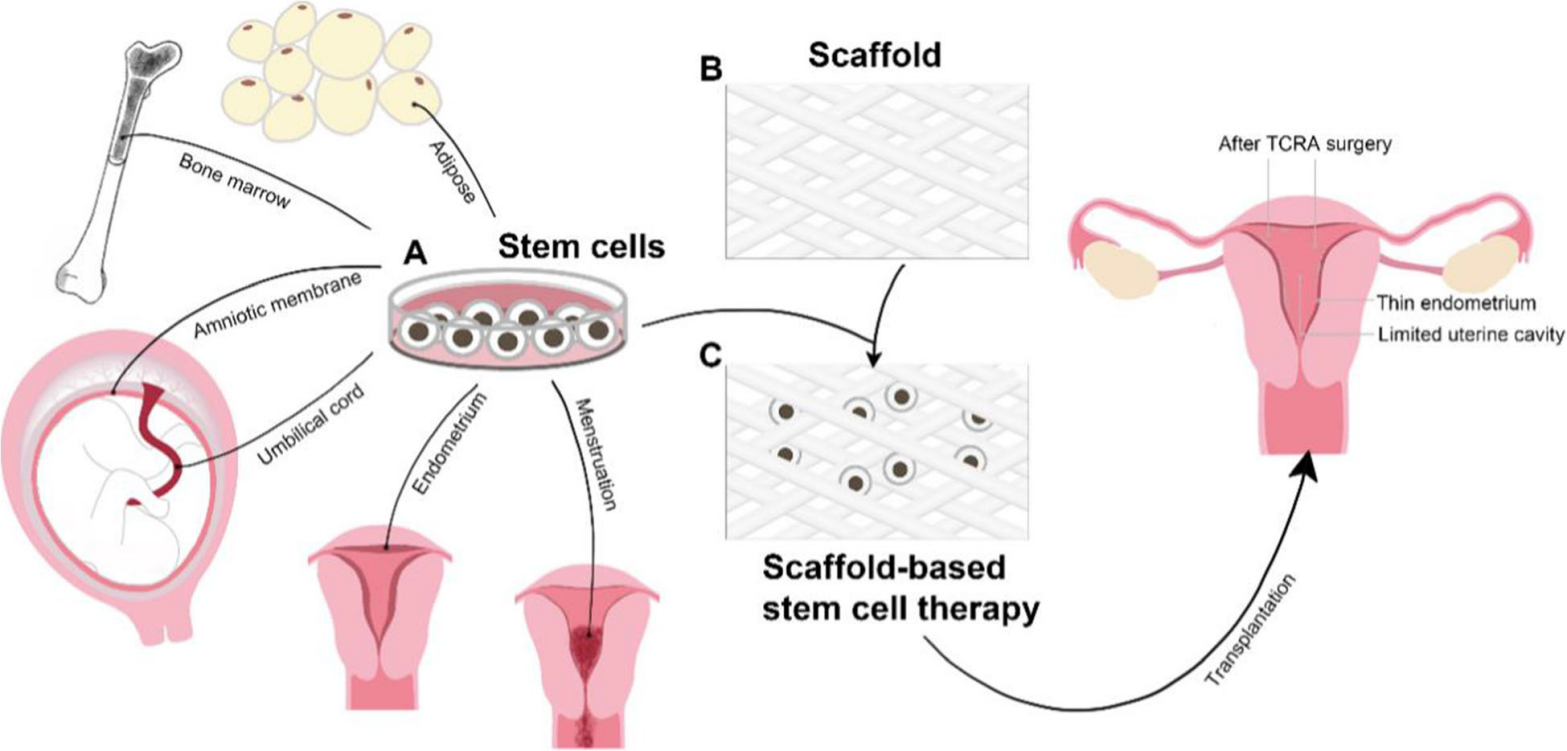
Scaffold-based cell/molecular delivery. (**A**) Stem cells derived from bone marrow (BMDSCs), human amniotic membrane (AMSCs), umbilical cord (UC-MSCs), adipose tissue (ADSCs), menstrual tissue (MenSCs) and endometrium (ESCs) can be transplanted postoperatively for IUA patients. (**B**) Certain scaffolds (such as collagen scaffolds and heparin-proloxamer scaffolds) carry stem cells to repair damage to the uterine wall as a potential system (**C**)

Recently, postoperative stem cell transplantation for IUA patients has been expected to be a breakthrough in the treatment and improvement of reproductive outcomes. Stem cells (Fig. 8A) derived from bone marrow (BMDSCs), human amniotic cells (AMSCs), adipose cells (ADSCs), menstrual cells (MenSCs), umbilical cord cells (UC-MSCs) and endometrium cells (ESCs) have the potential to improve endometrial repair. There are some problems and limitations of stem cell transplantation; for example, certain cytokines secreted by stem cells may stimulate angiogenesis. The effectiveness and safety as well as the specific molecular mechanism remain to be clarified [78].

Stem cells have high self-renewal and multidirectional differentiation potential and can be amplified in vitro. Moreover, stem cells possess the ability of “horizontal differentiation” or “cross-line differentiation”. Thus, stem cell-based treatment methods have become ideal candidates for tissue engineering research. For patients with IUAs or endometrial injury, there have been many studies on whether the differentiation potential of stem cells can be used to regenerate damaged endometrium. Zhang *al.* found that BMSCs could differentiate into endometrial epithelial cells when cocultured with endometrial stromal cells [94]. Du and Bratincsak also demonstrated that BMSCs could be differentiated into endometrial stromal cells and endometrial epithelial cells [95, 96]. These studies all proved the feasibility of the differentiation of stem cells into endometrioid cells and provided an experimental basis for the application of stem cell therapy after endometrial injury. Angiogenesis is an essential link in the repair of endometrial injury. Investigations have also revealed that stem cells have the ability to approach and migrate to damaged tissue [95, 97]. When the vascular structure is damaged, ischaemia and hypoxia lead to tissue cell necrosis. Appropriate stem cells can differentiate into blood vessel cells, promote and accelerate the formation of new blood vessels in the damaged area, and thus promote tissue repair.

#### Secreting a variety of bioactive molecules to promote the recovery of damaged cells and inhibit inflammation

Transplanted stem cells can produce cytokines by autocrine and paracrine action after entering the damaged site. Exosomes secreted by haemopoietic stem cells were found to upregulate anti-apoptotic genes (BCL2L1, BCL2 and BIRC8) and downregulate pro-apoptotic genes (CASP1, CASP8 and LTA), thus inhibiting apoptosis of damaged cells and playing an important role in tissue repair and regeneration [98]. When tissue damage is serious, endogenous stem cells are not enough to maintain homeostasis of the internal environment. Therefore, exogenous stem cells should be transplanted to promote angiogenesis and tissue repair.

### Lacking of immunogenicity and immunoregulation method

Recently, the possibility of stem cells modulating the immune response has attracted extensive attention. Transplanted homologous stem cells can evade immune surveillance by the recipient [99], and introduction of allogenic stem cells will effectively treat severe acute graft-versus-host disease that is insensitive to immunosuppressive agents [100]. The cytokine microenvironment is the most important factor affecting the immune regulation of stem cells; for example, high levels of γ-interferon (IFN-) secretion can protect stem cells from NK cells [101]. Previous studies have demonstrated that the Toll-like receptor (TLR) signalling pathway of stem cells may affect their differentiation, proliferation, migration and immunosuppression functions [102]. On the one hand, TLRs may promote the immunosuppressive ability of stem cells, thus limiting the extent and scope of tissue damage and contributing to the remission of inflammation; on the other hand, TLR-activated stem cells may secrete a variety of inflammatory cytokines to reverse the suppression of the immune response and restore immunity after the remission of inflammation.

### Polymer based delivery systems

#### Collagen-based scaffolds

Collagen is a biodegradable material with good compatibility and rarely causes allergic reactions. Currently, collagen-based scaffolds (Fig. 8B) have been widely used in clinical applications, including promoting skin, bone and nerve repair [74]. For mild-to-moderate IUAs, a pure collagen scaffold itself can be used as a physical barrier to prevent IUA postoperative adhesion (Fig. 8D). The reason may be that collagen scaffolds possess degradability, good histocompatibility, and low inflammatory reactions. It does not need to be removed after uterine cavity operation. The reported studies showed that after the establishment of a rat model of full-thickness uterine injury, cytokines or stem cells attached by collagen scaffold transplantation (Fig. 8C) promoted the regeneration of endometrium and myometrium and improved the pregnancy outcome of rats [75–77, 103].

**Fig. 9:**
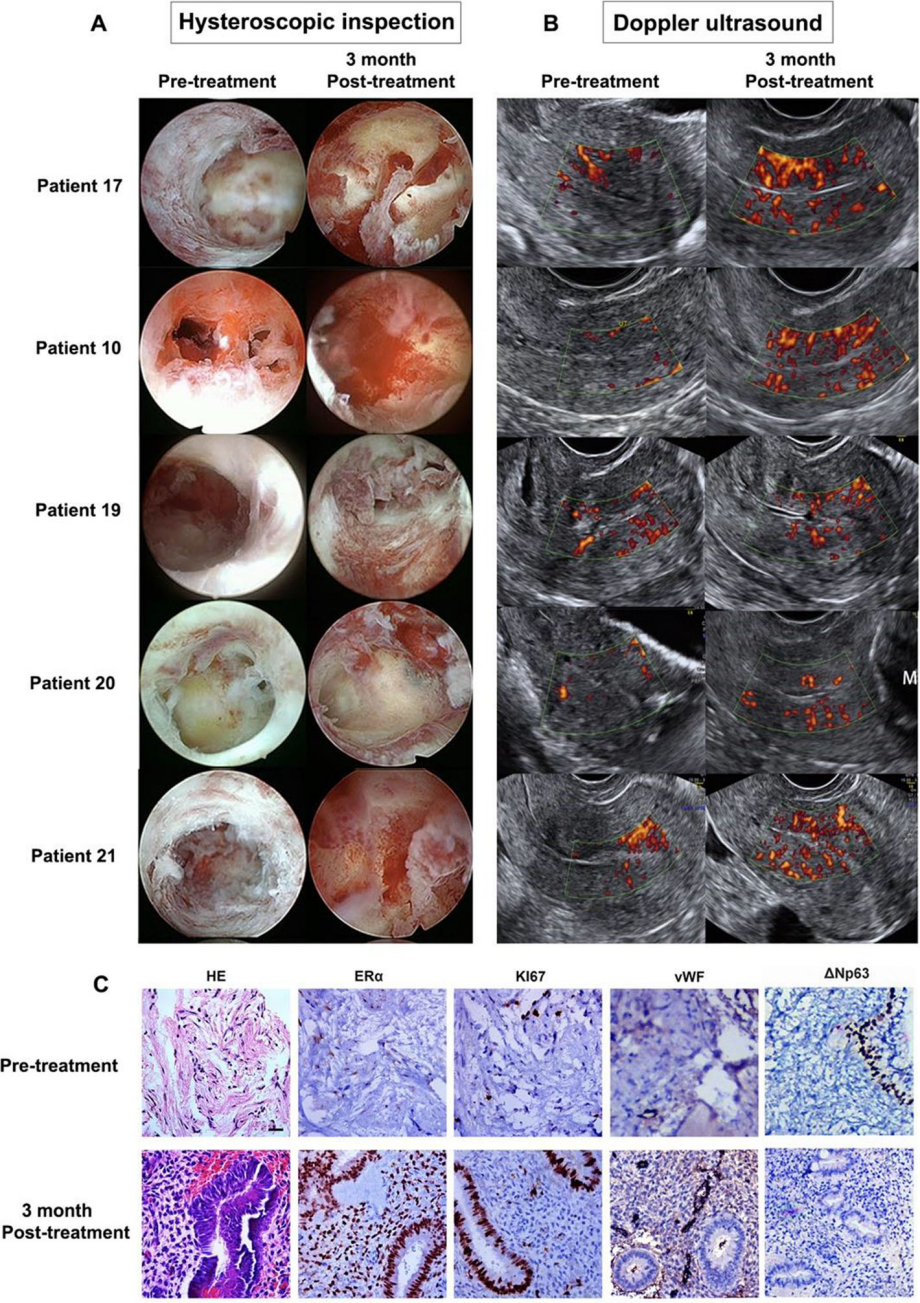
Umbilical cord mesenchymal stem cells (UC-MSCs) based on collagen scaffolds were applied for patients with recurrent intrauterine adhesion [104]. (**A**) Hysteroscopic inspection images and (**B**) blood flow by Doppler ultrasound from the same 5 patients before and after UC-MSC/collagen treatment. (**C**) Immunohistochemical staining of ERα, Ki67, vWF and ΔNp63 in endometrial biopsy samples obtained from patients before and after UC-MSC/collagen treatment. Bar: 100 μm. ERα oestrogen receptor alpha, vWF von Willebrand factor. Reprinted with permission from Ref. [104]

Collagen scaffolds can also carry stem cells or therapeutic bioactive factors to repair damage to the uterine wall. In vivo experiments of full-thickness defects of the rat uterine wall demonstrated that collagen scaffolds combined with bFGF were used as therapeutics and improved regeneration abilities and vascularization [103]. Scar remodelling caused by degradation of scar collagen, neovascularization and rapid vascularization can be observed [76]. More recently, BMDSCs and embryonic stem cells combined with collagen scaffolds have been used to repair full-thickness damage to the rat uterine wall [75, 77]. Human UC-MSCs supported by collagen scaffolds in the treatment of recurrent intrauterine adhesion have entered phase 1 clinical trials **(**Fig. 9**)** [104]. It has been reported that drug/stem cell-collagen therapeutic systems could promote endometrial hyperplasia and repair and effectively improve pregnancy outcome from the perspective of structure and function [78, 105]. This work produced porous collagen scaffolds with controlled lyophilization [106]. The organoids were directed by the scaffold and integrated with primary endometrial stromal cells. With endometrial organoids on the surface, a multicellular model was formed, and both stromal and epithelial cells were stimulated by hormones, resulting in stromal decidualization and epithelial differentiation. A prospective, uncontrolled, phase I clinical trial including 26 patients with recurrent IUA-induced infertility was followed up for 30 months [104]. During the operation, 1 × 10^7^ UC-MSCs were loaded on collagen scaffolds and transplanted into the uterine cavity. Three months after the operation, the mean maximum thickness of the endometrium increased, and the score of intrauterine adhesion decreased compared with that before the treatment. Histology revealed that MSC/collagen scaffold treatment improved endometrial proliferation, differentiation, and neovascularization therapy. The regenerated endometrium contained only patient DNA by the result of DNA short tandem repeat (STR) analysis. At the end of 30 months of follow-up, 10 of the 26 patients were pregnant. Therefore, UC-MSC-loaded biodegradable collagen scaffolds could be transplanted into the uterine cavity and demonstrated a safe and effective treatment method for IUA.

UC-MSCs possess the advantages of low immunogenicity and proliferation potential. Collagen scaffolds loaded with UC-MSCs (CS/UC-MSCs) were designed for endometrial regeneration [107]. CS/UC-MSC transplantation maintained the normal lumen structure and promoted endometrial regeneration and collagen remodelling. Subsequently, endometrial endogenous cell proliferation and epithelial cell recovery were induced. The expression of oestrogen and progesterone receptors was reasonably enhanced. Therefore, local application of CS/UC-MSCs can prevent re-adhesion, promote endometrial regeneration, and improve pregnancy outcomes in patients with severe IUAs.

**Fig. 10:**
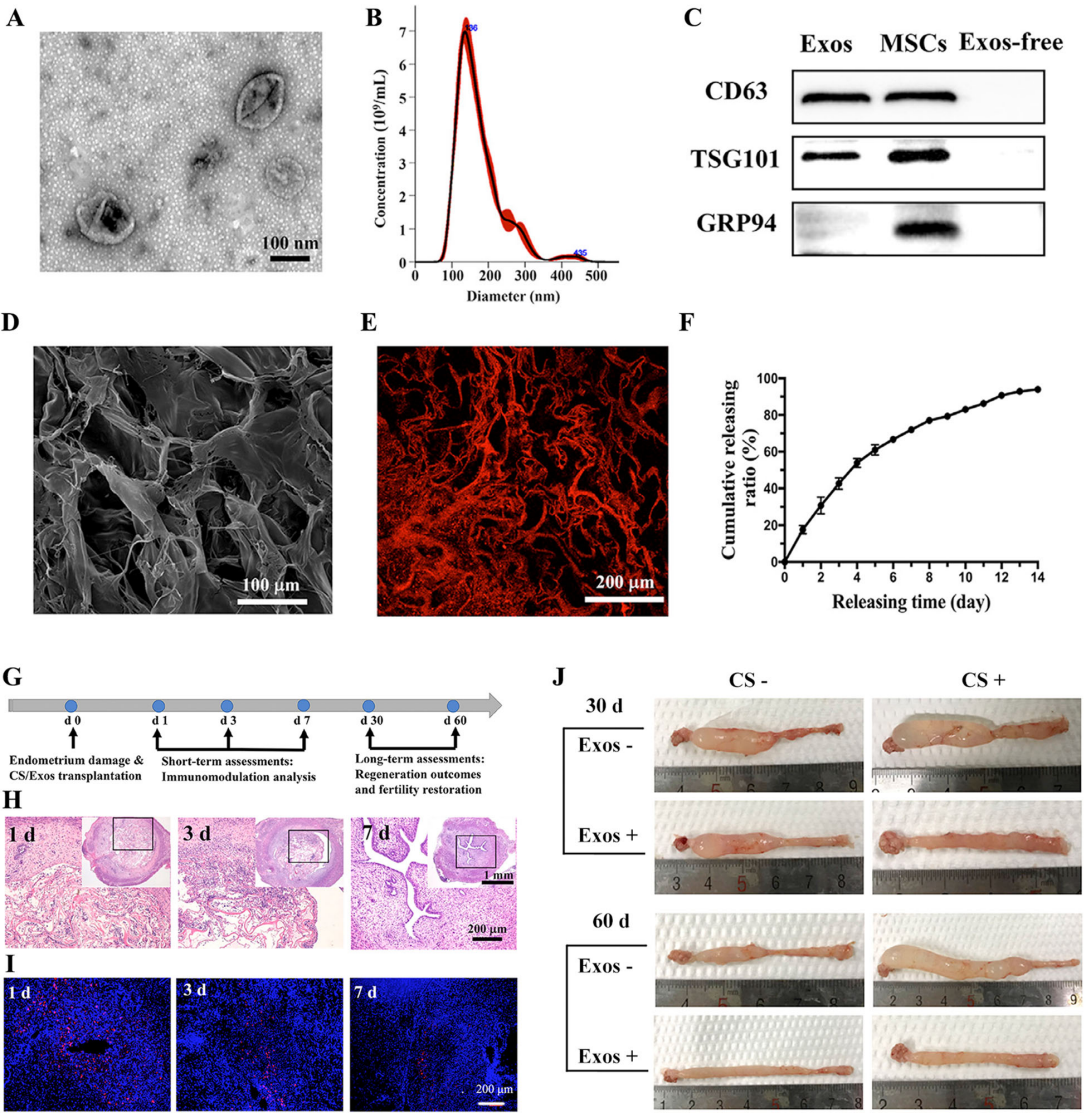
A construct of exosomes and collagen scaffolds (CS/Exos) and its ability to regenerate endometrium [108]. (**A**) Representative transmission electron microscopy (TEM) image. (**B**) Size distribution profile of exosomes derived from umbilical cord mesenchymal stem cells (UC-MSCs). (**C**) Western blot analysis of CD63, TSG101, and GRP94 in exosomes (Exos), UC-MSCs and exosome-free conditioned medium (Exos-free). (**D**) Scanning electron microscope (SEM) image of CS/Exos. (**E**) Representative confocal laser scanning microscopy (CLSM) image of CS/Dil-labelled Exos. (**F**) The release profile of exosomes from CS/Exos. (**G**) Schematic diagram of in vivo experiments. (**H**) Tracing of implanted CS/Exos in vivo by HE staining. Inserts are the corresponding overview pictures at a lower magnification, and the magnified regions are marked with black squares. (**I**) Tracing of implanted CS/Dil-labelled Exos in vivo by immunofluorescent staining. (**J**) Morphologies of uteri under different treatments for 30 and 60 days. Reprinted with permission from Ref. [108]

Stem cell-based delivery therapeutic strategies have proven some promising results in the treatment of IUAs. However, the potential tumorigenicity and low infusion limit further application. More recently, exosomes derived from stem cells exhibited similar functions to those derived from cells. Hence, exosomes and collagen scaffolds (CS/Exos) could be designed and constructed for endometrial regeneration **(**Fig. 10**)** [108]. CS/Exo transplantation significantly induced endometrial regeneration and collagen remodelling. The expression of oestrogen receptor/progesterone receptor was increased and used to restore fertility. MiRNAs enriched in exosomes are the main mediators of exosome-induced macrophage polarization. The construction of CS/Exos promotes the polarization of M2 macrophages, reduces the inflammatory response and increases the anti-inflammatory response. Therefore, this work highlights the therapeutic prospects of CS/Exos and their translational application in the treatment of severe IUAs.

#### Heparin - Proloxamer

Heparin-proloxamer 407 (HP) is a new kind of polymer material with low toxicity and high affinity. This material not only possesses the anticoagulant and thrombolytic properties of heparin but can also be used to combine temperature-sensitive characteristics. HP has been widely used for loading therapeutics and released with sustained behaviour. The copolymers consist of hydrophobic polypropylene oxide (PPO) and hydrophilic polyethylene oxide (PEO) units. With the variation of concentration and temperature, the aggregation of PPO and PEO can form the micellar corona and core, respectively. As a result, these self-assembled micelles in ordered cubic or hexagonal phases could produce thermosensitive hydrogels [84, 85]. Heparin-poloxamer hydrogel (HP hydrogel) is a degradable and nontoxic bioactive material that can also be applied for sustained drug release. Injection of a 17β-oestradiol heparin-poloxamer thermosensitive hydrogel (E2-HP hydrogel) into the uterine cavity of IUD rats promoted the regeneration of damaged endometrium and inhibited cell apoptosis. E2-HP hydrogels may activate the ERK1/2 and MAPK p38 pathways and then upregulate the expression of kisspeptin [86]. Furthermore, the hydrogels can serve as an effective supporting matrix to prevent IUAs after endometrial injury, which can also be considered a delivery system for the controlled release of proper drugs. However, due to the rapid turnover of endometrial mucus, the retention and malabsorption of therapeutic drugs in the damaged endometrial cavity are two important factors that may hinder the pharmacological effects.

A mucoadhesive hydrogel was prepared by using heparin-modified poloxamer (HP) as the matrix material, combined with keratinocyte growth factor (KGF) to cure the IUA [87]. The rheological and mucoadhesive properties of the mucoadhesive hydrogel (EPL-HP) can be easily controlled by the functional excipient ε-polylysine (EPL). Meanwhile, EPL can significantly enhance the proliferation of endometrial epithelial cells and angiogenesis in the regenerated endometrium in vitro, which implies that EPL-HP hydrogels delivered with KGF may be another promising approach to treat damaged endometrium.

Similarly, the combination of vitamin C (Vc) with PF-127-encapsulated BMSCs exhibited better endometrial restoration of IUA rats [109]. 17β-Oestradiol (E_2_) has been commonly used after TCRA in IUA patients. However, the concentrations in the injured endometrium are relatively low due to poor solubility. Micelles of heparin-poloxamer-encapsulated E_2_ were used as a thermosensitive hydrogel (E_2_-HP hydrogel) to sustain the release of E_2_ [88]. Injection of E_2_-HP hydrogel in IUA rats showed a prolonged and sustained release of E_2_ at the injured sites and more effective regeneration of endometrium, which might be due to the suppression of endoplasmic reticulum (ER) stress-related apoptosis via the activation of PI3K/Akt and ERK1/2 signalling pathways. To design an E_2_ delivery system with ideal solubility, control release and suitable intrauterine moulding. E_2_ was encapsulated in nanoparticulate decellularized uterus (E_2_@uECMNPs), and then E_2_@uECMNPs were embedded into the thermosensitive aloe-poloxamer hydrogel (E_2_@uECMNPs/AP) as multiple components. Administration of E_2_@uECMNPs/AP hydrogel promoted cell proliferation and inhibited cell apoptosis, thus enhancing endometrial regeneration in a rat model [89].

## Tissue engineering based methods

### Cell sheet engineering

Stem cell transplantation is a promising method to promote endometrial tissue regeneration in IUA therapy. Traditional methods of injecting cells directly into the uterine cavity, where the stem cells cannot stay long enough since the mucus is secreted and drained rapidly to grow and develop. A variety of scaffolds made from natural or synthetic polymers provide appropriate culture conditions for cell growth and tissue formation. This makes scaffold-based cell/tissue delivery a constantly developing approach for endometrial regeneration and the primary system for cell therapy. However, there are still serious limitations, especially in cell-intensive tissue engineering. “Cell sheet engineering”, a scaffold-free tissue technology that has recently emerged in the fields of tissue engineering and regenerative medicine. Since Okano proposed cell membrane technology [110], it has been developed to obtain the endogenous cell membrane structure formed by cells and extracellular matrix by adding active factors such as vitamin C into ordinary culture medium for a period of continuous cell culture. Compared with exogenous scaffold materials, the cell membrane does not need enzyme digestion and can improve the cell survival rate and reduce cell loss. Dense membranes formed by the extracellular matrix and cells secreted by cells themselves effectively repair tissue damage. Based on the above advantages, cell membrane technology as a new cell transplantation method has been widely used in the field of regenerative medicine [111, 112].

**Fig. 11:**
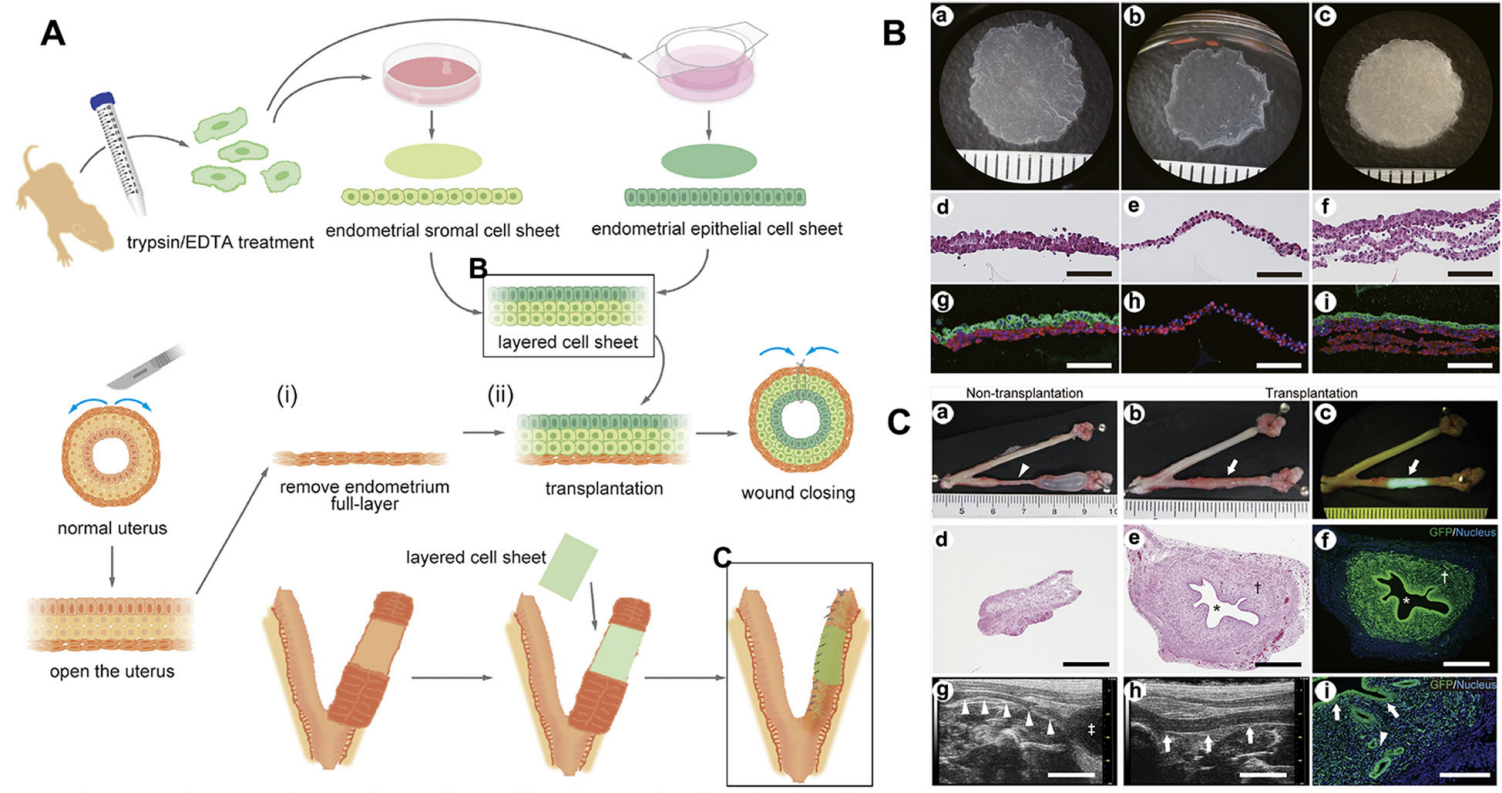
Endometrial regeneration using cell sheet transplantation techniques in rats facilitates successful fertilization and pregnancy [113]. (**A**) Scheme for preparation of the full-thickness endometrium defect model and cell sheet transplantation. Isolated GFP-positive endometrial cells were separated, and two types of confluent cell cultures (stromal fraction and epithelial fraction) were harvested as contiguous cell sheets. (**B**) Macroscopic observations and histological analyses with H&E staining and immunostaining of endometrial cell sheets. (a–c) Endometrial epithelial cell sheet produced from syngeneic rat primary endometrial cell harvests (a), rat endometrial stromal cell sheet (b), and three-layer cell sheet assembled from one epithelial cell sheet and two stromal cell sheets (c). (d–i) Histological analysis of each cell sheet by HE staining (d–f), CK-18 (green) and vimentin (red) immunostaining (g–i). (**C**) Effects of endometrial cell sheet transplantation. (a–c) Macroscopic observations of resected uteri without fluorescence (a, b) and with fluorescence (c). (d, e) Histological analysis of the uterine cross section by HE staining and (f, i) GFP immunostaining. (g, h) Ultrasound image of uteri post-surgery. Reprinted with permission from Ref. [113]

A cell sheet, a tissue-like cell monolayer, is fabricated by the surface grafting of thermoresponsive poly(N-isopropylacrylamide). This approach can deliver this cell-dense tissue to the target site effectively. Cell sheet transplantation techniques in rats can regenerate endometrial tissue with confirmed histological structure and normal physiological function to support fertilization and pregnancy (Fig. 11) [113]. In addition, thermoresponsive poly(N-isopropylacrylamide)-grafted surfaces enable the fabrication of a tissue-like cell monolayer, a “cell sheet”, and can efficiently deliver this cell-dense tissue to damaged sites without the use of scaffolds. For example, adipose-derived stem cells (ADSCs) have been applied as seed cells to form cell sheets without scaffolds. The 3D ADSC sheet was transplanted into the uterine cavity of IUA rats. ADSCs were mostly detected in the basal layer of the regenerated endometrium at 21 days after transplantation of ADSC sheets, some of which differentiated into endometrial stromal cells [114].

Specifically, novel cell sheet-based tissue engineering includes vascularization for scaled-up 3D tissue constructs, induced pluripotent stem (iPS) cell technology for cell sheet fabrication and microfabrication for arranging tissue microstructures. With thermal manipulation, the cell sheet is expected to develop diverse 3D cell/tissue models in regeneration medicine, tissue modelling and drug delivery [115]. Therefore, there is a prospective future for cell sheet engineering in restoring IUAs.

### Three-dimensional (3D) printing scaffold-based tissue engineering

**Fig. 12:**
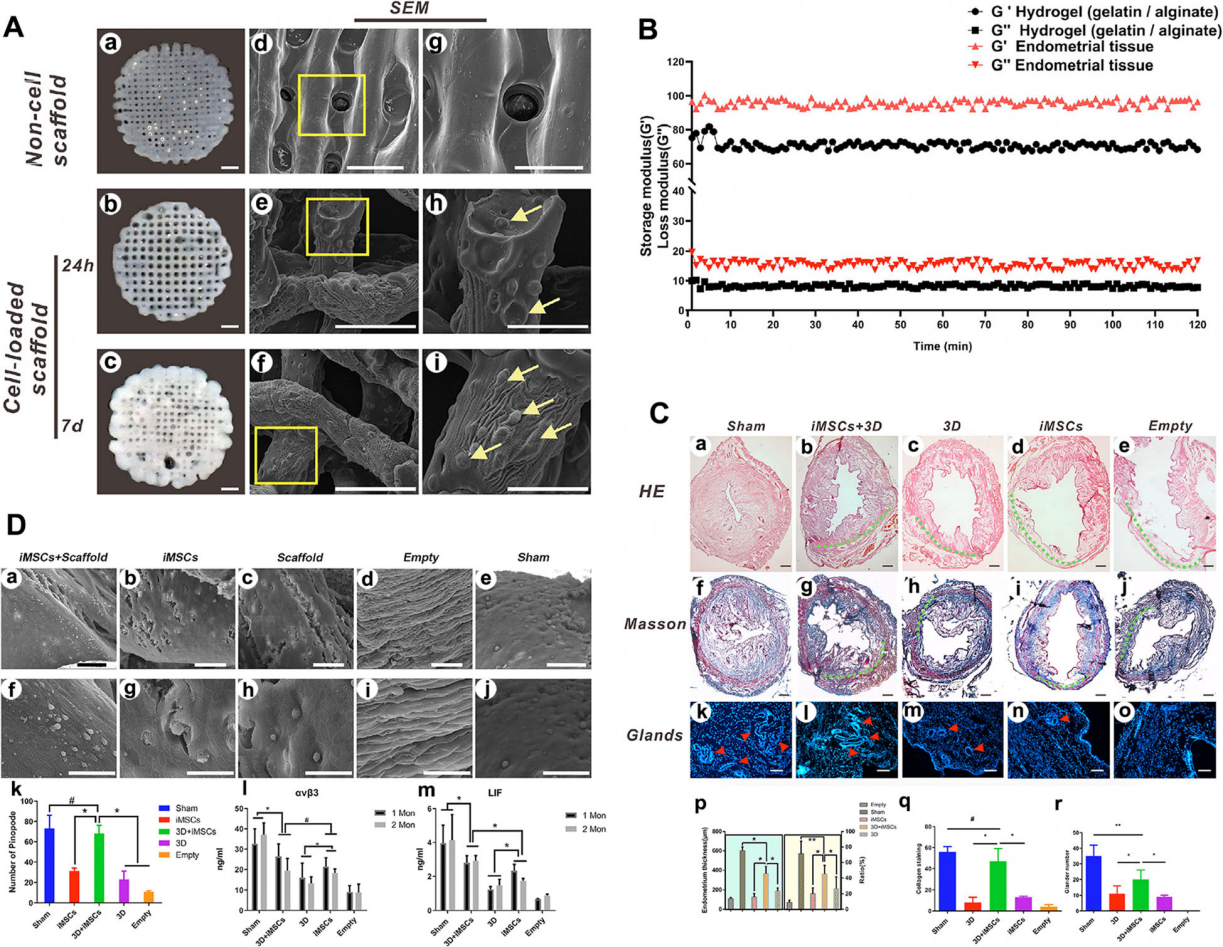
3D Bioprinting a human induced pluripotent stem cell (iPSC)-derived mesenchymal stem cell (hiMSC)-loaded scaffold for repair of the uterine endometrium [116]. Characterization of the 3D-printed hydrogel scaffold and iMSC-loaded hydrogel scaffold. (**A**) Observation of the cell-free hydrogel scaffold (a) and the cell-loaded hydrogel scaffold (b: 24 h after printing; c: 7 days of culture after printing). (**B**) Mechanical properties of the bioink hydrogel scaffold compared with those of endometrial tissue over the 120-min test period. (**C**) Morphological assessment of the regenerated endometrium. (**D**) Receptivity analysis of the regenerated endometrium: (a–j) Scanning electron microscope (SEM) images and (k) quantification of pinopodes on the surface of the regenerated endometrium on the fifth day after conception in animals from all the groups after endometrial regeneration. (l, m) ELISA results at 15 days and 1 month after surgery. Reprinted with permission from Ref. [116]

Currently, tissue engineering is developing to prepare 3D scaffolds that are more clinically compatible. With the development of 3D printing technology, 3D scaffolds have made greater development in tissue engineering. Titanium, −tricalcium phosphate, polylactic acid and other materials could be prepared by 3D printing technology. 3D printing is a kind of material-making technology to obtain accurate shapes with the help of 3D CT data, which is able to produce special shapes and achieve a more ideal effect in repairing tissue defects. 3D cultures mimic the extracellular matrix (ECM) structure and function, and 3D stents are suitable to provide mechanical support for cells and promote tissue regeneration [81]. Contemporarily, 3D scaffolds have attracted increasing attention in the field of tissue engineering. Notably, scaffolds made by the combination of 3D printing **(**Fig. 12**)** [116] and electrospinning techniques may be the most promising approach for preventing intrauterine adhesions and promoting endometrial regeneration [117].

**Fig. 13:**
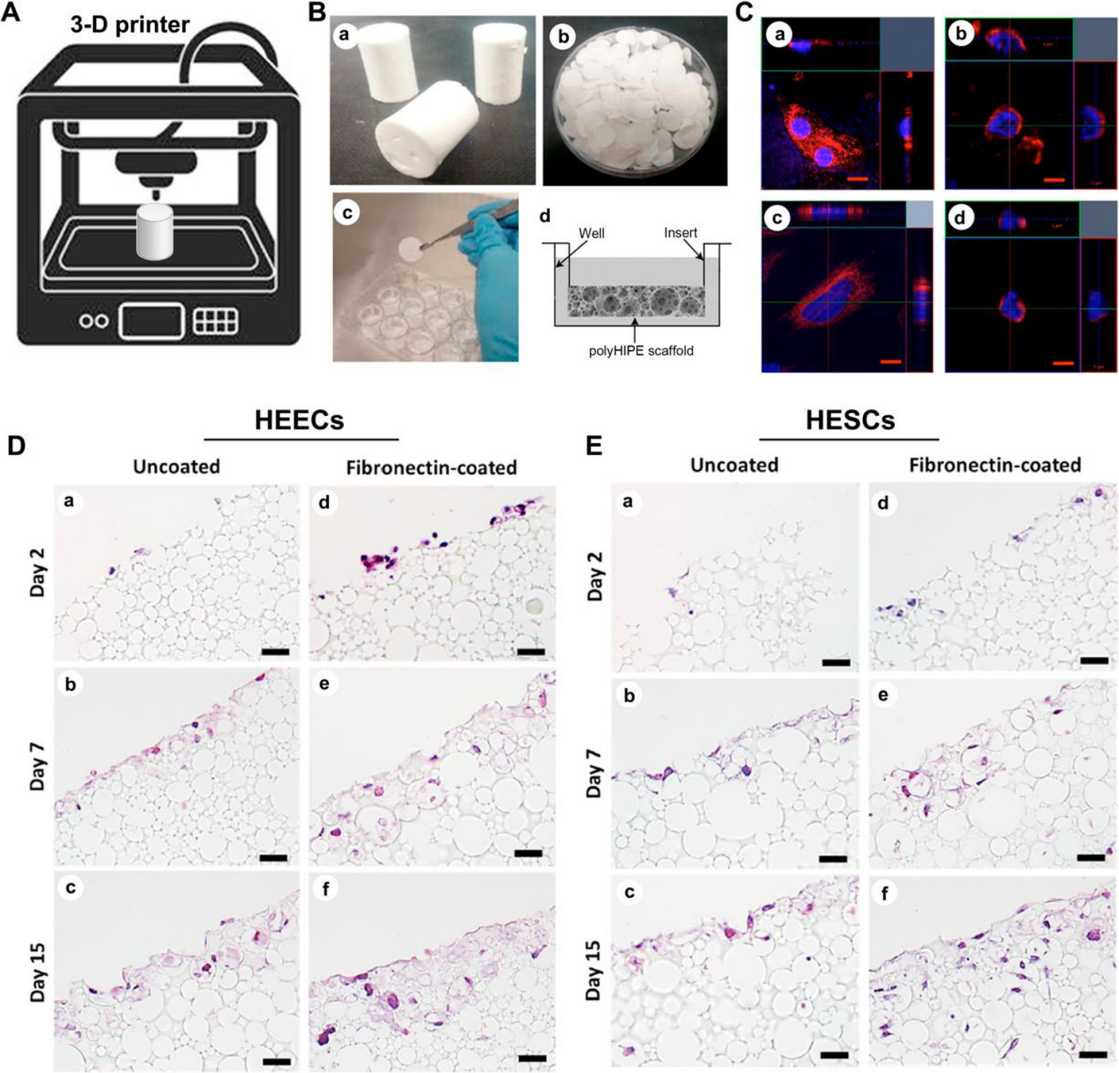
Enhanced differentiation potential of primary human endometrial cells cultured on three-dimensional (3D) scaffolds [118]. (**A**) The schematic diagram of the 3-D printer. (**B**) (a) Photographs of polyHIPE monoliths, (b) discs of 400 μm thickness, and (c) inserted in the cell culture well plate. (d) Presentation of a fully submerged polyHIPE scaffold in culture media using a Transwell insert. (**C**) Laser scanning confocal micrographs of cultured HEECs (a) in 2D; (b) in polyHIPE scaffold and HESCs (c) in 2D; and (d) in polyHIPE scaffold. Immunofluorescence staining of HEECs by anti-CK18 antibody/DAPI and HESCs by antivimentin antibody/DAPI. Bars: 5 μm. HE staining of HEECs (**D**) and HESCs (E) growth in polyHIPE scaffolds using 24-well plate format: (a, d) Day 2; (b, e) Day 7; (c, f) Day 15. Scaffolds were either left uncoated (a − c) or coated with fibronectin 5 (d − f). Fibronectin coating modestly promotes cell adhesion and migration into the material. Bars: 50 μm. Reprinted with permission from Ref. [118]

Conventional 2D monolayer cultures of epithelial and stromal cells fail to approximate the complex 3D architecture of natural human endometrial tissue. A study used emulsified templated porous polyps (known as polyHIPEs) as a scaffold for the incubation of primary endometrial epithelial cells and stromal cells (HEECs and HESCs) (Fig. 13) [118]. After the detection of histology, morphology and RNA sequencing, the polyHIPE scaffold ideally simulated the structure, function and microenvironment of the human endometrium. The PolyHIPE scaffold is considered to be a promising model for the study of tissue cells in vitro, which is especially suitable for the study of female intrauterine adhesion and infertility. An amine-reactive N-hydroxysuccinimide ester and a photoactivatable nitrophenyl azide, N-sulfosuccinimidyl-6-(4′-azido-2′-nitrophenylamino) hexanoate (sulfo-SANPAH), are linked and utilized to functionalize polyHIPE surfaces, which can be used to conjugate various compounds or biomolecules. In a study of IUA therapy, fibronectin-conjugated polyHIPE scaffolds promoted the attachment and function of primary human endometrial stromal cells [119].

However, the research and development of 3D scaffold materials still face the following problems. First, the production cost is expensive. Thus, personalized application promotion is difficult. Finally, the optimum porosity and pore size for different materials to repair defects still cannot reach consensus. Research on the fusion of stem cells and 3D printing technology of modified scaffold materials is still insufficient; how to realize the vascularization of 3D scaffold materials needs further research. The addition of active factors into 3D scaffold materials to promote endometrial repair needs more research data to verify. The development of 3D stent materials in endometrial repair after IUA has broad prospects, although there are also great challenges.

### Electrospinning fibrous scaffold-based tissue engineering

**Fig. 14:**
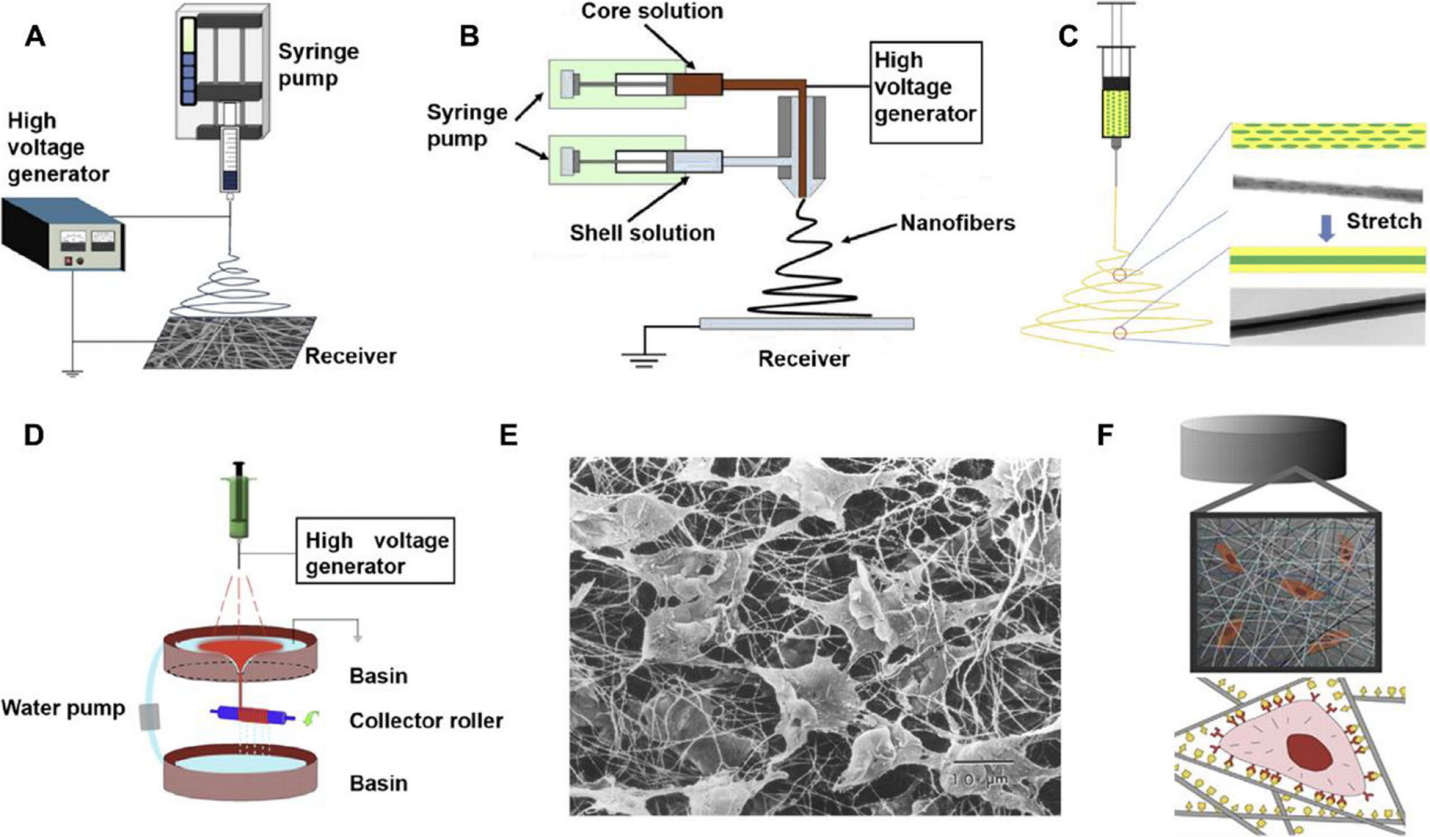
Electrospinning technique [123]. (**A**) Schematic of different electrospinning techniques. (**A**) Traditional electrospinning; (**B**) coaxial electrospinning; (**C**) emulsion electrospinning; (**D**) dynamic waterflow electrospinning. (**E**) Scanning electron microscopy (SEM) of fibroblasts in native tissue after freeze-drying. (**F**) Nanofibres have a greater surface area and therefore adsorb more protein, providing more adhesion sites for cell membrane receptors. Reprinted with permission from Ref. [123]

Electrospinning is a commonly used method to prepare fibrous structures ranging in diameter from microns to nanometres. Electrospinning works by adding high voltage electricity to a solution or melt of a polymer, which can be rapidly stretched to form a Taylor cone under high voltage electric field forces [120]. In particular, jets will be formed when the material is spun by overcoming the surface tension. Under a high voltage electric field force, these jet streams are stretched at high speed, the solvent evaporates rapidly, the jet solidifies and falls on the receiving device, and finally, the fibre mesh is formed. Electrospun nanofibres have unique advantages such as high porosity and large specific surface area. Recently, biocompatible and biodegradable polymer materials, such as cellulose acetate (CA), ethyl cellulose (EC), polylactic acid (PLA), collagen protein, collagen, etc.) by the electrospinning technique was applied to prepare micro/nanostructure nanofibres for wound dressings, tissue engineering, slow drug release, energy application, and other fields [121]. Nanofibre scaffolds prepared by electrospinning can be used to provide an extracellular matrix environment for the cells [81, 122]. With a high surface area, the electrospun nanofibres can absorb more protein, providing more adhesion sites for cell membrane receptors **(**Fig. 14**)** [123]. Therefore, nanofibrous electrospun scaffolds are considered promising tissue-engineered scaffolds due to their high porosity, interconnection between the pores, reduced friction and increased surface area-to-volume ratio, which can magnify cell-scaffold interactions [124].

### Droplet microfluidics technology based porous scaffolds

Microfluidic is a technology that processes or manipulates tiny fluids in microchannels (tens to hundreds of microns in size), providing precise control over the fluids. Through the design of clipped microfluidic chip and the principle of single/multiphase flow control in microscale space, the droplet template with uniform size, neat and orderly arrangement can be formed [125]. Therefore, the three-dimensional ordered porous tissue engineering scaffold based on microfluidic technology possesses obvious advantages, which may be a technology for an ideal uterine stent. Recently, biocompatible polymer materials, such as GelMA and sodium alginate, combined with the microfluidic technology to prepare the three-dimensional porous scaffold [69]. The scaffold possesses good biocompatibility, swelling and degradability, and can quickly restore its original shape after deformation. Meanwhile, the open porous structure connected the inside and outside gives the scaffold broad space and specific surface area, which can be used as an excellent carrier for drug and cell transportation. In addition to the simple physical prevention of intrauterine adhesions, this drug-loaded porous scaffold system can slowly release drugs in the injured area and further promote the repair of endometrium.

## Conclusion and prospect

In conclusion, IUA, or Asherman’s syndrome, is a common and recurrent gynaecological disease with menstrual disorders and reproductive dysfunction that seriously affects the physiological and psychological health of female reproduction. Hysteroscopy has evolved into the gold standard for the diagnosis of IUAs by virtue of its accuracy and intuitiveness, and TCRA is the first choice for the treatment of IUAs, supplemented by hormones and other medications to improve menstrual volume. The objective is to restore the normal uterine cavity and place an intrauterine barrier after surgery. Significant achievements have been made to maintain a physiological structure and eliminate defects over the decades, while complete restoration of the functional endometrium is still a problem. Previous studies have pointed out the promising roles of bioactive materials in enhancing endometrial healing and fertility outcomes.

Recently, some forms of biological barriers, such as amniotic membrane, urinary bladder matrix (UBM), platelet-rich plasma (PRP) and small intestine submucosa (SIS), have demonstrated significant potential in the treatment of IUAs. Some biomaterial-based barriers, containing many functional stem cells and cytokines, can provide powerful conditions for endometrial repair, proliferation and growth. Meanwhile, the anatomical structure of the uterine cavity will be maintained. On the other hand, polymer-based materials such as hyaluronic acid, chitosan, and PLGA are also ideal degradable biomaterials and can be processed into bioactive scaffolds for IUA treatment. More recently, proper stem cell transplantation has proven to be a new breakthrough in IUA treatment. These scaffolds combined with repairing functional stem cells can promote the treatment efficiency for IUAs. Previous studies of animal tests and clinical trials have both demonstrated the ideal effect on promoting endometrial repair and growth and late pregnancy, with insignificant side effects. Therefore, emerging biomaterial fabrication technologies have been extensively applied in clinical and other interdisciplinary fields.

This review systematically describes cell sheet-, 3D printing- and electrospinning scaffold-based therapeutic strategies for IUA treatment applications. However, there are still many problems in the application of these engineered bioactive materials. The single institution, small sample size, selection bias, unquantifiable factors and other factors have limited the research process in IUA treatment. Due to inconsistencies in evaluation criteria and the lack of randomized controlled trials with large sample sizes, it has been difficult to fully evaluate the effectiveness of various adjuvant therapies after TCRA. Therefore, the evidence on all the approaches we discussed above in clinical practice is still limited, and proper clinical solutions to maximize endometrial repair and fertility promotion are still needed. Hence, the effective treatment of IUAs is still a tough and hot research area. Restoration of a functional endometrium is essential for successful reproductive outcomes. Well-designed clinical trials are needed to determine the most appropriate diagnostic and therapeutic modalities.

Although considerable progress in the treatment of IUA has been obtained, relevant studies of the molecular mechanisms have rarely been reported. Besides, there is still no safe and efficient therapy to prevent the recurrence. To date, comprehensive treatment by hysteroscopy has been the mainstream treatment method. Other fashionable IUA treatment approaches, including hormone therapy, stem cells, and other emerging new clinical methods, have been explored in IUAs therapy. However, none of the above methods have been proven to acquire the desired achievements in clinical treatment.

Recent research has proven that bioscaffolds combined with therapeutic drug and stem cells for local intrauterine administration have been demonstrated good results in the treatment of IUAs compared with systemic injection manner. Bioscaffolds are good for endometrial cells attachment and proliferation and the local delivery of therapeutic stem cells or drug molecules to facilitate endometrial repair. However, the biocompatibility and biodegradability of the implanted bioscaffolds are key concerns that must be considered. Accompanying advances in biomaterials, it is desirable to develop bioscaffold-based carriers with additional functionalities for IUA management. Meanwhile, to enhance the efficacy of stem cells or therapeutic molecules, the composition, preparation technology and modification of bioscaffolds must been carefully investigated. Reconstruction of the biomimetic repair microenvironment is also an important indicator for IUAs. Thus, a novel preparative technique should be developed to construct specific structural bioscaffolds. Additional performance, such as binding affinity, mechanical properties, and low immunogenicity, can favour the transportation of therapeutic drugs or stem cells. Currently, the specific standardization of the construction of therapeutic bioscaffolds for the prevention of IUAs is still inadequate. Therefore, future exploration may focus on developing multimodal treatment strategies to combine therapeutic drugs or stem cells and then deliver them to targeted site and eventual release the therapeutic factors in a control way, which may be thinkable way to solve the current clinical IUA diseases.

## Data Availability

Not applicable.
